# Prediction of COVID-19 hospitalisation, ICU admission or death following ChAdOx1 vaccination using artificial intelligence: A clinical predictive model from the English RAVEN study

**DOI:** 10.1371/journal.pone.0336449

**Published:** 2026-02-20

**Authors:** Anshul Thakur, Bernardo Meza-Torres, Xuejuan Fan, Rachel Byford, Mark Joy, Wilhelmine Meeraus, Sudhir Venkatesan, Sylvia Taylor, Simon de Lusignan, David A. Clifton

**Affiliations:** 1 Institute of Biomedical Engineering, University of Oxford, Oxford, United Kingdom; 2 Nuffield Department of Primary Care Health Sciences, University of Oxford, Oxford, United Kingdom; 3 Medical Evidence, Vaccines & Immune Therapies, AstraZeneca, Cambridge, United Kingdom; 4 Medical and Payer Evidence Statistics, Biopharmaceuticals Medical, AstraZeneca, Cambridge, United Kingdom; 5 Royal College of General Practitioners (RCGP) Research and Surveillance Centre (RSC), London, United Kingdom; Sheikh Hasina National Institute of Burn & Plastic Surgery, BANGLADESH

## Abstract

**Objectives:**

This study identifies predictors of severe COVID-19 following completion of two-dose primary series of the AZD1222 COVID-19 vaccine, employing eXtreme Gradient Boosting (XGBoost) and Shapely additive explanations (SHAP), as an explainable artificial intelligence (AI) approach.

**Method:**

A retrospective cohort study using linked primary care data from the Oxford-Royal College of General Practitioners Clinical Informatics Digital Hub (ORCHID), including computerised medical records of over 19 million people in England, for the period from 8th December 2020–31st December 2021, as part of the Real-world effectiveness of the AZD1222 COVID-19 vaccine in England (RAVEN) study. We evaluated a two-dose primary series of the AZD1222 vaccine on COVID-19 related hospitalisation, ICU admission or death.

**Results:**

A total of 4,515,280 individuals with a two-dose primary series of AZD1222 vaccine were analysed, where 7,171 individuals had a record of severe COVID-19. Variables with the greatest predictive weight for COVID-19 mortality in vaccinated individuals were age ≥ 85 years, high Cambridge Multi-Morbidity Score, and chronic heart, respiratory and kidney diseases; variables predicting COVID-19 hospitalisation following completed primary series included high CMMS, obesity, and being offered early COVID-19 vaccination in the national vaccine campaign (e.g., vaccinated during the first quarter of 2021); predictors of COVID-19 ICU admission included obesity, female sex, being offered early COVID-19 vaccination in the national vaccine campaign, chronic kidney disease and diabetes. Across models, age ≥ 85 years was highly predictive of mortality and moderately predictive of hospitalisation. However, for ICU admission it was reported as not predictive.

**Conclusion:**

Obesity, chronic heart, respiratory and kidney diseases were the main predictors across models, which is comparable to the scientific literature, validating the explainable AI approach. XGBoost can accurately predict severe outcomes in fully vaccinated individuals. Predictive models built on real-world primary care data can help to timely identify individuals to be prioritised for vaccination booster.

## Introduction

The UK was one of the first countries that introduced a mass vaccination campaign for COVID- 19 and first started vaccinating the elderly population [1]. Vaccination with the ChAdOx1 vaccine (AZD1222) started in early January 2021. Real-world evidence demonstrated the effectiveness of two-dose primary-effectiveness against hospitalisation due to SARS-CoV-2 variants including Alpha, Delta, and Omicron, similar to that with mRNA vaccines as well as durability of protection over 4–6 months against hospitalisation and death [2–5]. The Real-world effectiveness of the Oxford/AstraZeneca COVID-19 vaccine in England (RAVEN) study was initiated in March 2021 to assess the effectiveness of the AZD1222 vaccine. As the need for booster COVID-19 vaccination was anticipated [6], and was introduced in the UK in September 2021, it was recognised that the RAVEN study could also evaluate extended objectives, such as identifying the predictors of severe COVID-19 in fully vaccinated individuals.

A sub-optimal response in individuals post COVID-19 vaccination is defined as a severe SARS-CoV-2 infection despite being fully vaccinated with two doses. Individuals with suboptimal responses have been reported to have lower seroconversion after booster vaccination, [7–9] and to include high-risk groups such as those with immunosuppressive conditions, multimorbidity, or older age groups [10]. Large-scale real-world routine datasets, based on electronic health records (EHRs), may allow for the identification of previously unreported risk groups. Due to their sample size, data heterogeneity and the potential linkage to multimodal data sources, they may be particularly suited for the implementation clinical predictive models based on explainable artificial intelligence (AI) [11]. Explainable AI refers to methods aiming to understand the reasoning behind AI predictions, for example by coupling predictive modelling methods (e.g. XGBoost), to techniques rendering more intuitive explanative outputs, such as Shapely additive explanations (SHAP). While conventional statistical methods, such as regression analysis, allow for adjustment for known confounders, explainable AI adds an emphasis on clinical predictive value. The combination of such conventional and AI approaches, may render a potentially useful combination for targeting public health interventions. Prompt identification of suboptimal responders may allow for early vaccination boosters or other mitigation strategies to ensure the optimal protection of vulnerable populations in the face of emerging viral threats.

Explainable AI and machine learning (ML) methods have been used for predicting side effects, disease severity, and mortality in COVID-19 [12–14]. However, to the best of our knowledge there is no investigation on the clinical predictors of severe outcomes in vaccinated individuals which could be targeted in future epidemiological emergencies. Given that severe outcomes such as mortality were highest amongst vulnerable populations, such as in long-term care and assisted living facilities [15,16] which the AZD1222 vaccine was designed to address, it is imperative to understand predictors of severe events, as a proxy for suboptimal response, in individuals who received a two-dose primary series of AZD1222 vaccination.

## Aim and objectives

The aim was to investigate predictors of severe COVID-19 in individuals after receiving a two-dose primary series of AZD1222 vaccine, where severe COVID-19 is defined as COVID-19 related hospitalisation, intensive care unit (ICU) admission, or death, by implementing clinical predictive modelling based on explainable AI. The study objective was to identify predictors of COVID-19 related hospitalisations, ICU admission, or death amongst those vaccinated with a two-dose primary series of AZD1222 during 2021.

## Materials and methods

### Study design

This was a retrospective cohort study using linked secondary data databases in England.

This study is based on the cohort used in the RAVEN vaccine effectiveness study. The RAVEN study design matched individuals who were vaccinated with one, two, or three doses of AZD1222 COVID-19 vaccine to unvaccinated individuals, and modelled the occurrence of the event of interest. The design included a rolling cohort matching approach, [17] in which matched unvaccinated individuals who subsequently become vaccinated were censored, along with their matched vaccinated counterpart. The newly vaccinated individual could then re-join the study within the vaccination group (provided they met the inclusion and exclusion criteria) and were matched to a new unvaccinated control.

For this analysis we focused on vaccinated individuals in the matched cohorts who received 2 doses of AZD1222 COVID-19 vaccine.

### Setting and data sources

The setting was England, using primary care electronic healthcare records from the Oxford-Royal College of GPs Clinical Informatics Digital Hub (ORCHID), a near real-time nationwide health-care dataset stored in secure Trusted Research Environment (TRE) [18,19].

We included data from approximately 19 million individuals from the ORCHID primary care database who were linked to secondary care and COVID-19 data. Individuals in ORCHID are broadly representative of the English population in terms of age, gender, ethnicity, NHS Region, socioeconomic status, obesity and smoking habit [20].

The study covers the period of time from first roll-out of COVID-19 vaccines in England to the end of December 2021.

Through the NHS DARS we requested access and linkage of ORCHID to secondary care data assets collected as part of routine care and commissioning activities in the NHS, including:

- National Immunisation Management Service (NIMS) – including datasets on COVID-19 Vaccination Status
- Hospital Episode Statistics (HES) – covering patients attending accident and emergency (A&E) units, admitted for treatment, or attending outpatient clinics at NHS hospitals in England, including details about length of stay
- Civil Registrations: Information including the date, place and certificated cause of death from the Office for National Statistics (ONS)
- The COVID-19 Second Generation Surveillance System (SGSS) – Demographic and diagnostic information from laboratory test reports for patients tested for COVID-19 in England only. It included the first positive results from pillar 1 (swab testing in Public Health England (PHE) and NHS hospital labs and pillar 2 (swab testing for the wider population) (Time lag – less than 1 week)
- COVID-19 UK Non-hospital Antigen Testing Results (Pillar 2) data includes a range of COVID19 test results, including National Pathology Exchange (NPEX). This is broadly similar to SGSS, but only covers Pillar 2 data, however, contains the full result set – i.e., all positive, negative and null results.

### Study population

The study population was individuals in England who received a two-dose primary series of AZD1222 COVID-19 vaccine between the 4^th^ January 2021 and the 31^st^ December 2021. Study participants were aged 18 years or over, considered eligible for vaccination by age at the time of conception of the study.

The following inclusion criteria were used:

- Eligible for vaccination by age (age 18 or over)
- Two doses of AZD1222 COVID-19 vaccination
- Be eligible for linkage with Second Generation Surveillance System (SGSS) and National Pathology Exchange (NPEX) to identify history of prior COVID-19 infection
- Have continuous data coverage in other linked databases, including ORCHID, for a minimum of 12 months prior to 4th January 2021 for assessment of baseline variables including socio-economic status, comorbidities, and follow-up of outcome events.

We excluded people with a history of COVID-19 infection (confirmed by reverse transcriptase polymerase chain reaction [RT-PCR]) prior to vaccination using the SARS-CoV-2 infection datasets, SGSS and NPEX.

Individuals with missing values were excluded to avoid the introduction of noise in the analysis which could have compromised the models as learned imputations are imperfect. The only variables which reported missing values were ethnicity, smoking status and BMI.

### Variables

#### Outcomes.

We studied the following outcomes when occurring 15 days after the second COVID-19 vaccine dose, and before any booster vaccine dose:

- COVID-19 related hospitalization: Including emergency inpatient admission. Defined as hospitalisation with an ICD-10 primary code for COVID-19 (U071, U072) on admission to hospital, i.e., DIAG01 in HES APC.
- COVID-19 related ICU admission: defined as an ICU admission during a hospitalisation for COVID-19 as defined above
- COVID-19 related death: where COVID-19 is the underlying cause of death, based on an ICD-10 code (U071, U072) for COVID-19 in ONS mortality data.

#### Exposures.

Exposure was defined as two doses of AZD1222 vaccine administered at least 15 days from each other, from 4^th^ January 2021 onwards.

We used data from the NIMS, containing information on the COVID-19 vaccine brand, date of vaccination, and dose sequence (first, second or booster dose).

#### Other covariates.

Covariates were investigated as potential predictors, defined using the most recent measurement 12 months prior to the vaccine roll out date – 4^th^ January 2021 for AZD1222 COVID-19 vaccine.

Sociodemographic characteristics included age, gender, ethnicity, NHS Region, socioeconomic status, and body mass index (BMI) category. Comorbidities were defined following the Greenbook Chapter 14a [21], and included chronic respiratory, kidney, heart, vascular, liver, and neurological diseases; diabetes mellitus, severe mental illness, morbid obesity, asplenia or spleen dysfunction, and immunosuppression due to disease or treatment. Composite scores included the Cambridge Multimorbidity Score quartile (CMMS, 1 = least comorbidity) [22], and the Electronic frailty score [23] category among individuals aged 65 years or older (Fit, mild, moderate severe, missing). We included prior Influenza vaccination (last 2 years), COVID-19 vaccine batch number, month of COVID-19 vaccination (calendar time), and long-term care (for people aged 70+). The propensity to consult (health seeking behaviour) was measured by the number of GP visits in year prior to the baseline date, coded as 0,1,2,3,4,5 + visits).

Covariates were obtained from EHRs using ORCHID and HES – linked data. The Care Quality Commission (CQC) register, was used to identify individuals under long-term assisted care.

### Statistical methods

#### Summary measures.

Descriptive statistics were used to describe demographic and clinical characteristics of individuals in the study cohort. Frequencies and percentages were used to describe categorical variables. Central tendency (mean or median) and dispersion (standard deviation or interquartile range) measures were used to describe continuous variables. Standardised mean differences were used to compare subgroups.

#### Predictive modelling.

A ML model using extreme gradient boosting trees (XGBoost) was trained to predict the study outcomes among individuals with a two-dose primary series of AZD1222 vaccine. An analysis was then performed on the trained model to identify variables that were positively associated with the positive model predictions.

##### Extreme gradient boosting trees:

XGBoost is a ML algorithm that obtains a predictive model by assembling multiple decision trees and is capable of modelling non-linear relationships between the predictors and the outcome. The output of the predictive model is a weighted sum of the prediction of decision trees. Gradient boosting performs empirical risk minimisation to minimize the deviation or loss between the true prediction and this weighted sum of individual decision trees’ predictions. Gradient boosting starts with a single decision tree and incrementally adds more decision trees in a greedy manner while performing the empirical risk minimisation.

We used an XGBoost classifier that consisted of 100 weak estimators or decision trees having a maximum depth of 3 and the log-loss loss function. Hyperparameter tuning was used to select these parameters by maximising the average AUROC across all five folds. The search spaces for the number of trees and maximum depth were limited to {10,100,250,500,1000} and {2,3,4,5,6,7,8,9,10}, respectively. The learning rate of 0.01 was used to train the models. The log-loss is a standard choice for binary classification/prediction tasks.

We used Shapely additive explanations (SHAP) to identify the most relevant features in XGBoost. SHAP is a game theory-based method to explain the contribution of each feature in a model prediction for an input example. The sign of a SHAP value indicates the positive or negative association with the model outcome. It is possible to aggregate these local or individual contributions in order to gain a better understanding of the overall importance of features and their association with the model outcome.

#### Model validation.

The area under the receiver operating characteristic curve (AUROC) was used to evaluate model performance. The model was trained in a five-fold cross-validation setup, where we used five runs, with each run using 80% data for training and 20% for testing [24].

The model was trained multiple times with different random seeds (random initialisations) to ensure that similar average performance (in terms of AUROC across five-fold cross-validation) and similar characteristics of sub-responders were obtained in each run.

All analyses were performed using Python and R programming languages [25].

#### Sensitivity analyses.

To assess variation attributable to the modelling methodology, ML models using logistic regression (LR), and a fully connected neural network (DNN) were trained to predict the suboptimal response cases exhibiting the study outcomes among individuals with a two-dose primary series. The same validation methods as above described were followed (S5 and S6 in S1 File).

#### Missing values.

People with implausible values were excluded from the main cohort prior to matching. Implausible values included vaccination dates earlier than the first vaccine rollout date, and outlier values of BMI after observing the empirical distribution.

For the AI modelling, individuals with missing values were excluded. Exclusion was preferred over ML-based imputations to avoid the introduction of noise in the analysis.

#### Ethics approval.

Retrospective pseudonymized routine data were used for this study. Data was extracted for the period between the 4^th^ January 2021 and the 31^st^ December 2021, including data from patients registered in practices belonging to the Oxford Royal College of General Practitioners Research and Surveillance Centre (RCGP RSC), who did not dissent from their data being used for research purposes at their primary care practice [26]. These data are held at the ORCHID Hub, a TRE [26,27] that meets the NHS Digital Data Security and Protection standards [28]. The data included only the covariates specified, and authors did not have access to information that could identify individual participants during or after data collection. The data collection stage, when data were accessed for research purposes, was between 23rd August 2021 and the 15th December 2022. Ethics approval was granted by the NHS Health Research Authority London – Bromley Research Ethics Committee on May 2021, with REC reference: 21/HRA/1971 and IRAS Project ID: 300259.

## Results

### Descriptive results

**Fig 1:**
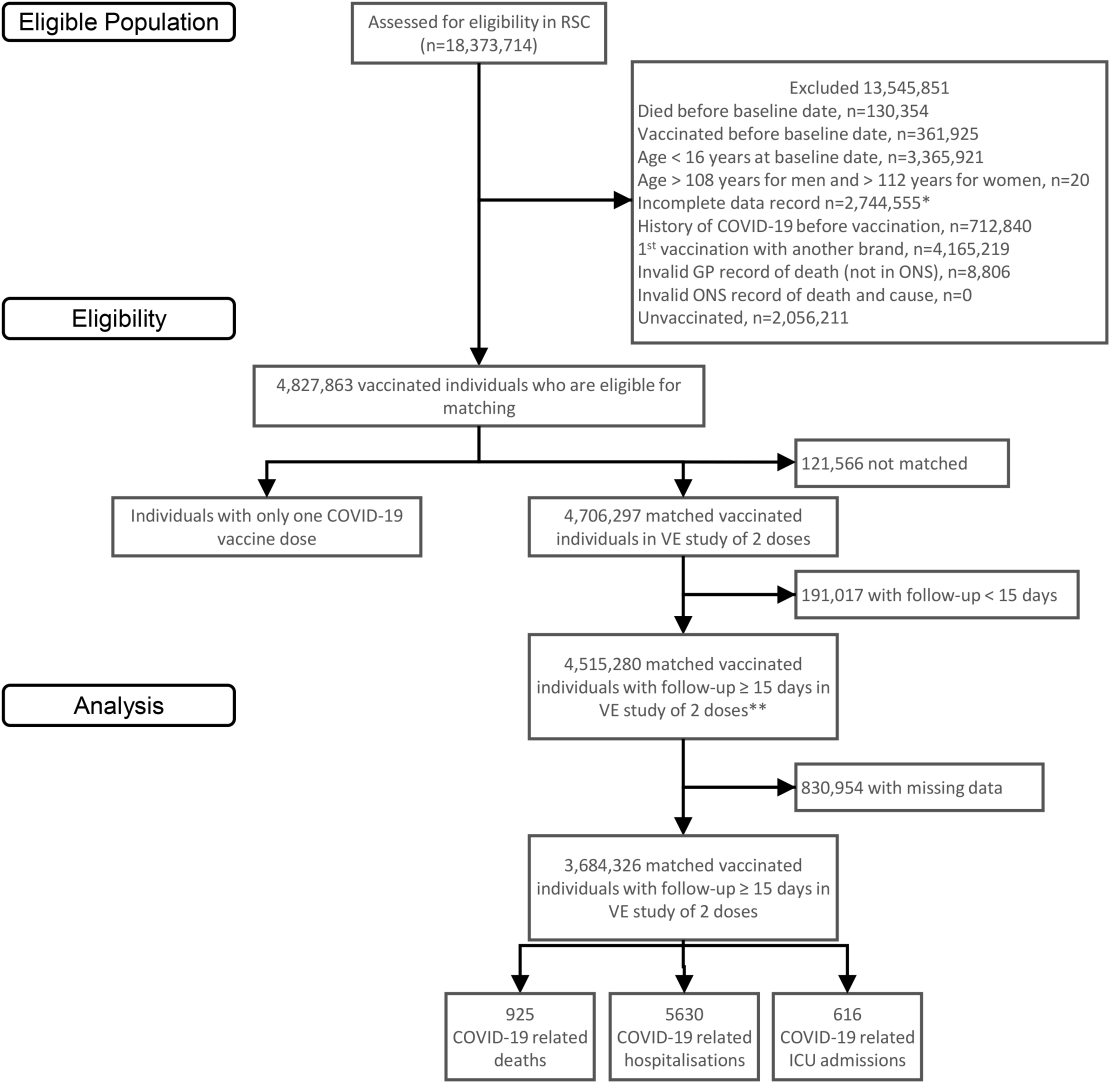
ORCHID cohort consort diagram. * Includes people who registered within a year of baseline, and those who registered over a year before baseline but deregistered at or in the 12 months before baseline, or between baseline and the 1st vaccination, or between their 1st and 2nd vaccination. VE – vaccine effectiveness; ONS – Office of National Statistics. **These are the full matched analysis cohorts used for the estimation of vaccine effectiveness for COVID-19 related death.

From over 18.3 million people with records in the ORCHID database, a total of 4,515,280 individuals had a record of a two-dose primary series of AZD1222 vaccine (Fig 1). After removing those with missing values 18.40% (n = 830,954), the study population comprised 3,684,326 individuals, with a mean age of 57.2 (SD = 14.0) years. Up to 52.5% (1,933,025) of the vaccinated cohort were female, and 47.5% (1,751,301) were male (Table 1).

**Table 1:** Participants included in the study cohort.

Variable	Category	All Vaccinated individuals	Vaccinated individuals After Removing Missing Values
Total		4515280	3684326
Age [mean (SD)]		56.5 (14.27)	57.2 (14.0)
Age category	16-24	97078 (2.1)	55595 (1.5)
	25-34	216788 (4.8)	164177 (4.5)
	35-39	173023 (3.8)	136877 (3.7)
	40-44	437921 (9.7)	345257 (9.4)
	45-49	511839 (11.3)	413206 (11.2)
	50-54	649420 (14.4)	525757 (14.3)
	55-59	631574 (13.9)	514851 (14.0)
	60-64	523665 (11.6)	434379 (11.8)
	65-69	422717 (9.4)	362115 (9.8)
	70-74	425677 (9.4)	369251 (10.0)
	75-79	248336 (5.5)	216286 (5.9)
	80-84	86846 (1.9)	73004 (2.0)
	85+	90396 (2)	73571 (2.0)
Sex	F	2292766 (50.8)	1933025 (52.5)
	M	2222514 (49.2)	1751301 (47.5)
Index of multiple deprivation quintile	1 (most)	667893 (14.8)	554972 (15.1)
	2	777425 (17.2)	634658 (17.2)
	3	910308 (20.2)	743230 (20.1)
	4	1001820 (22.2)	814331 (22.1)
	5 (least)	1157834 (25.6)	937135 (25.4)
Ethnicity	White	3462715 (76.7)	3292977 (89.4)
	Asian	245096 (5.4)	232231 (6.3)
	Black	96936 (2.1)	92549 (2.5)
	Mixed	37640 (0.8)	35228 (1.0)
	Other	34405 (0.8)	31341 (0.9)
	Missing	638488 (14.1)	–
BMI	Underweight (BMI < 18.5)	77659 (1.7)	61945 (1.7)
	Normal (BMI 18.5–24.9)	1404342 (31.1)	1211724 (32.9)
	Overweight (BMI 25–29.9)	1519686 (33.7)	1335947 (36.3)
	Obese (BMI 30–39.9)	1040128 (23)	917531 (24.9)
	Severe obesity (BMI 40+)	163833 (3.6)	144313 (3.9)
	Not recorded	293248 (6.5)	–
Smoking status	Never smoked	2476716 (54.9)	2047261 (55.6)
	Active smoker	691025 (15.3)	551516 (15.0)
	Ex-smoker	1283674 (28.4)	1085549 (29.5)
	Not recorded	63865 (1.4)	–
Region	East of England	311710 (6.9)	250012 (6.8)
	London	561672 (12.4)	495417 (13.4)
	Midlands	776494 (17.2)	648285 (17.6)
	North East and Yorkshire	534869 (11.8)	429594 (11.7)
	North West	705911 (15.6)	587838 (16.0)
	South East	990937 (21.9)	789698 (21.4)
	South West	633687 (14)	483482 (13.1)
Index month (month of second AZD1222 vaccination)	Jan-21	14 (0.0003)	10 (0.0002)
	Feb-21	1368 (0.03)	1106 (0.03)
	Mar-21	148369 (3.3)	123538 (3.4)
	Apr-21	1160236 (25.7)	998005 (27.1)
	May-21	1653042 (36.6)	1359492 (36.9)
	Jun-21	1157572 (25.6)	903795 (24.5)
	Jul-21	333367 (7.4)	252406 (6.6)
	Aug-21	43571 (1.0)	32252 (0.9)
	Sep-21	8204 (0.18)	6314 (0.17)
	Oct-21	4279 (0.09)	3323 (0.09)
	Nov-21	3445 (0.07)	2683 (0.07)
	Dec-21	1813 (0.04)	1402 (0.04)
Cambridge multimorbidity score quartile*	1 (lowest)	996552 (22.1)	804396 (21.8)
	2	935229 (20.7)	722086 (20.0)
	3	935375 (20.7)	735556 (20.0)
	4 (highest)	1648124 (36.5)	1422288 (38.6)
Comorbidities	Chronic respiratory disease	184211 (4.1)	165349 (4.5)
	Chronic kidney disease	201946 (4.5)	178181 (4.8)
	Chronic heart disease	567419 (12.6)	490497 (13.3)
	Chronic liver disease	109110 (2.4)	95687 (2.6)
	Chronic neurological disease	302526 (6.7)	254401 (6.9)
	Diabetes	382453 (8.5)	345943 (9.3)
	Severe mental illness	60866 (1.3)	54849 (1.5)
	Asplenia	30642 (0.67)	26021 (0.7)
	Immunosuppression	128513 (2.8)	111308 (3.0)
Flu vaccination (Current Season)		2353899 (52.1)	2122785 (50.9)

BMI – body mass index; CMMS – Cambridge multimorbidity score; EFI – electronic fragility index; N – number; NA – not applicable; SD – standard deviation

### Outcome data

A total of 7,171 (1.2%; 7171/602659) individuals had a record for severe COVID-19 during the study period. This included COVID-19 related deaths in 12.9% (925/7171), hospitalisations in 78.5% (5630/7171) and ICU admissions in 8.6% (616/7171) of cases (Table 2).

**Table 2:** Number of recorded COVID-19 related deaths, hospitalisation and ICU admission amongst individuals with a two-dose primary series of the AZD1222 vaccine in the ORCHID cohort (n = 7,171).

Outcome categories	Frequencies (%)
COVID-19 related deaths	925 (12.9)
COVID-19 related hospitalization	5630 (78.5)
COVID-19 related ICU admission	616 (8.6)

### Predictors of mortality, hospitalisation and ICU admission using trained XGBoost models and SHAP

#### Hospitalisation prediction.

**Fig 2:**
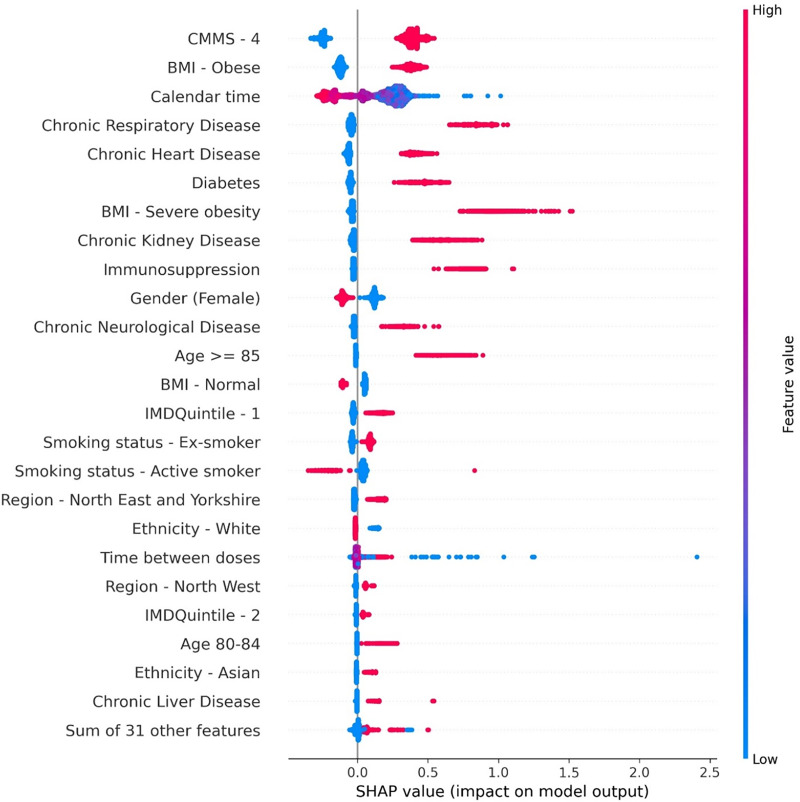
Beeswarm plots depicting top 25 most relevant features in XG Boost trained for COVID-19 hospitalisation prediction.

People with high CMMS score, obesity, chronic respiratory, heart and kidney disease, diabetes, immunosuppression, age ≥ 85 years and male sex are at a higher risk of a suboptimal response resulting in a COVID-19 related hospitalisation according to the SHAP values of the trained XGBoost model (Fig 2 and S11 in S1 File).

**Fig 3:**
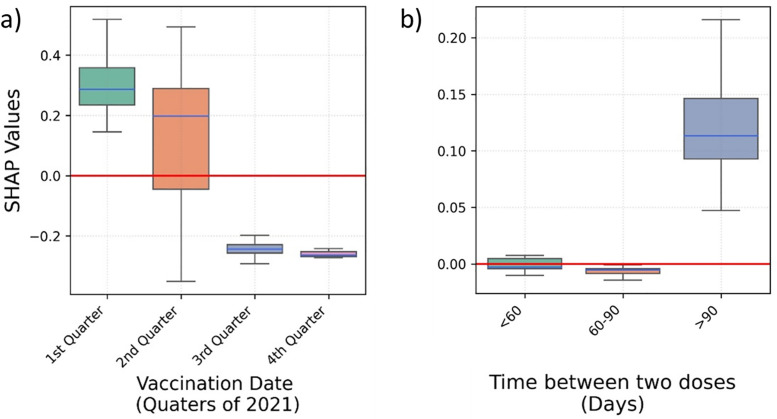
Box plots depicting the SHAP values predicting COVID-19 hospitalisation for a) the second vaccination date, and b) the time between two COVID-19 vaccination doses.

People who were vaccinated earlier in the year – first quarter of 2021 – were at higher risk of hospitalisation (Fig 3a). A larger gap between the two vaccine doses is positively associated with hospitalisation (Fig 3b); noting that time between vaccinations in the UK was defined as 11–12 weeks at the start of the vaccination campaign; later, in May 2021, it was reduced to 8–9 weeks.

**Fig 4:**
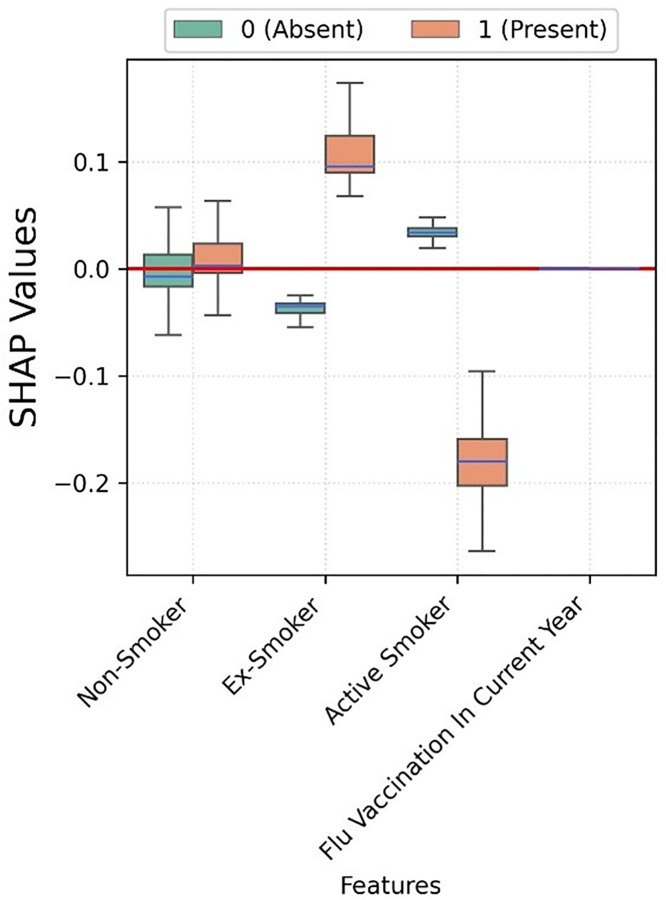
Box plots depicting the SHAP values for each category of smoking and flu vaccination status corresponding to XGBoost trained for COVID-19 hospitalisation prediction.

Flu vaccination seems to have no impact on the hospitalisation risk (Fig 4). Moreover, active smoking seems to have a negative association with hospitalisation.

#### Predicting ICU admissions.

**Fig 5:**
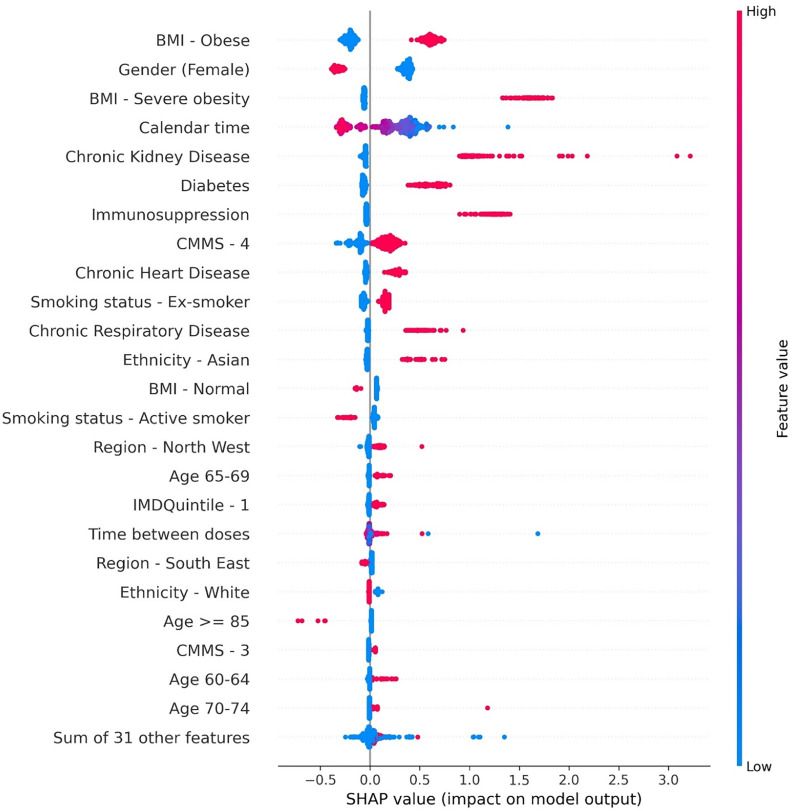
Beeswarm plots depicting top 25 most relevant features in XG Boost trained for COVID-19 ICU admission prediction.

Similarly as for the other two study outcomes, obesity, female sex, calendar time, chronic kidney disease, diabetes, and immunosuppression are associated to COVID-19 related ICU admission according to SHAP values from the trained XGBoost (Fig 5 and S12 in S1 File). Age ≥ 85 was negatively associated with ICU admission.

**Fig 6:**
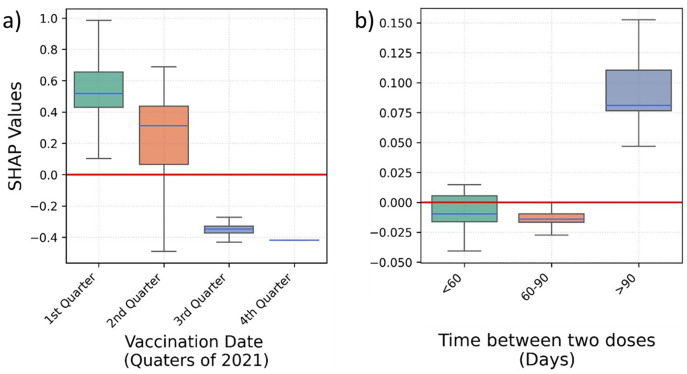
Box plots depicting the SHAP values predicting COVID-19 ICU admission for a) the second vaccination date, and b) the time between two COVID-19 vaccination doses.

Vaccination in the first two quarters of 2021, and a gap of over 90 days between vaccine doses are associated to higher risk of ICU admission (Fig 6).

**Fig 7:**
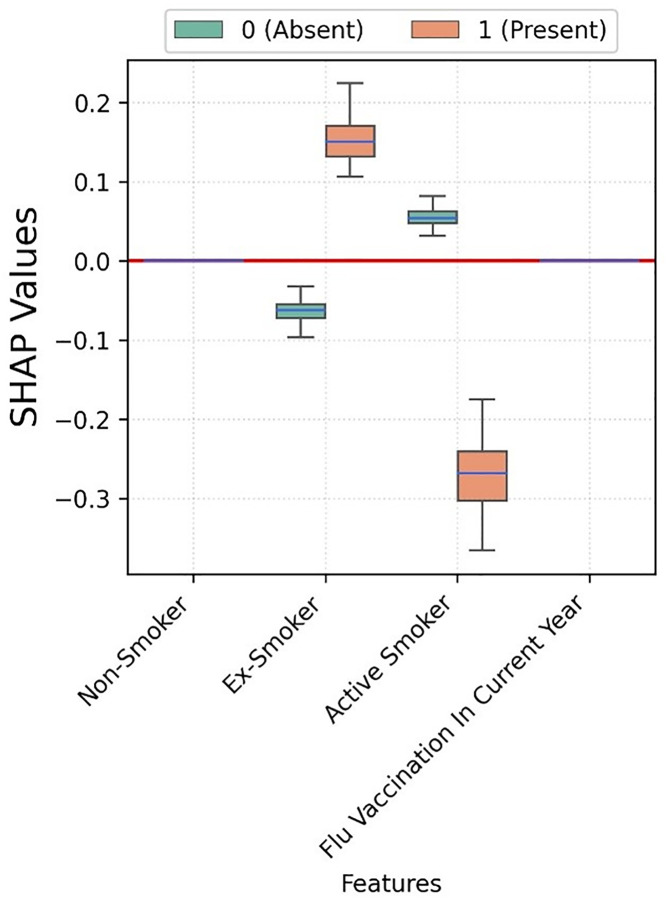
Box plots depicting the SHAP values for each category of smoking and flu vaccination status corresponding to XGBoost trained for COVID-19 ICU admission prediction.

Flu vaccination seems to have no impact on ICU admission risk, while active smoking is negatively associated (Fig 7).

#### Mortality prediction.

**Fig 8:**
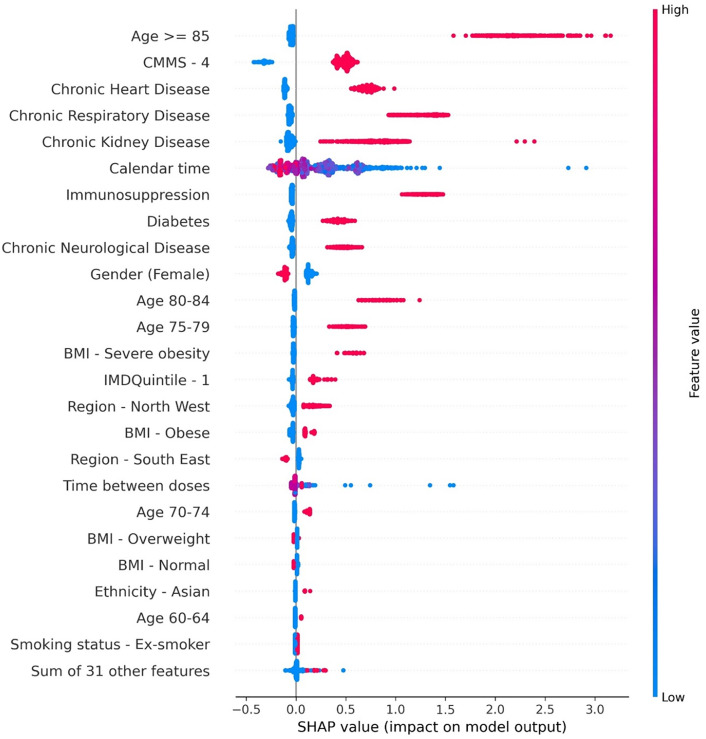
Beeswarm plots depicting top 25 most relevant features in XG Boost trained for COVID-19 mortality prediction. Figure Legend: Beeswarm plot exhibiting the impact of each variable of an input or subject on the outcome obtained from the XGBoost model. The y-axis represents the features, and the x-axis describes the SHAP value or impact on the model output. A SHAP value of 0 means that the feature has no impact on the model, while a positive value implies that a feature is positively contributing to the outcome of interest, and vice-versa. Each subject or input is represented by a single dot on each feature row. Multiple dots represent the density of SHAP values for that feature. The colour of dots for each feature represents the value of corresponding feature, providing a third dimension for the analysis. The colormap on the right side presents the feature magnitude. For discrete variables, the blue implies 0 and the red implies 1.

Age ≥ 85 years, high CMMS, chronic heart, respiratory and kidney disease were the variables with the highest SHAP values predicting cases resulting in COVID-19 related mortality for the XGBoost trained model. Males (Sex = 0) have a higher mortality risk than females. Immunosuppression, obesity, older age groups (80–84 years), and people who got vaccinated earlier in the year (calendar time) were also associated to higher mortality (Fig 8 and S10 in S1 File).

**Fig 9:**
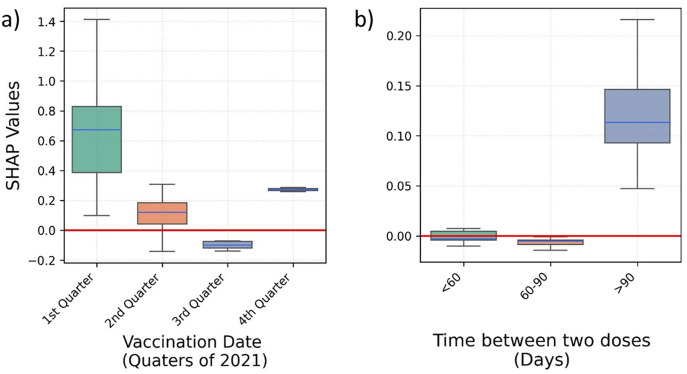
Box plots depicting the SHAP values predicting COVID-19 mortality for a) the second vaccination date, and b) the time between two COVID-19 vaccination doses. *Colours of boxes do not convey any specific information.

A higher risk of mortality is observed for a second vaccination dose received in the first quarter of the year 2021 (Fig 9a). Similarly, the larger the gap between two doses is positively associated with mortality (Fig 9b).

**Fig 10:**
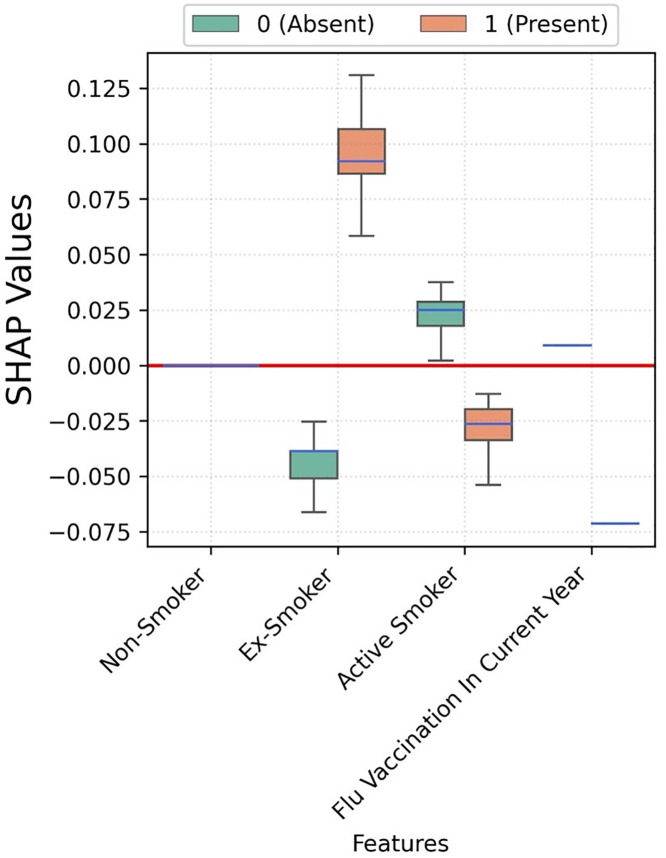
Boxplots depicting the SHAP values for smoking category and flu vaccination status corresponding to XGBoost trained for COVID-19 mortality prediction. Figure legend: Individuals who not belonging to a category and vice-versa are represented with 0 (Absent). Features such as Non-Smoker with no impact on model exhibit zero SHAP values. Some boxes degenerate to median line if there is no variance in SHAP values, e.g., Flu vaccination.

The SHAP values for smoking status and flu vaccination are small, and not relevant as predictors (Fig 10). However, flu vaccination was associated to a lower risk of mortality; while being ex-smoker was negatively associated. Active smokers do not seem to be associated to mortality as the spread contains both negative and positive SHAP values.

## Discussion

### Key results

#### Clinical predictors.

In the predictive modelling with XGBoost, age ≥ 85 years, high CMMS, and chronic heart, respiratory and kidney diseases were the variables with the greatest weight in predicting sub-optimal responses resulting in COVID-19 related mortality occurring at least 15 days after vaccination. For COVID-19 hospitalisation as an outcome, high CMMS, obesity, and being offered early COVID-19 vaccination in the national vaccine campaign (e.g., vaccinated during the first quarter of 2021) were found predictive in the model. Predictors of ICU admission included obesity, female sex, vaccination in the first quarter of 2021 (calendar time), chronic kidney disease and diabetes.

For unvaccinated individuals, the literature reports age as the strongest risk factor for severe COVID-19, with a mortality risk ratio (RR) >7 for those aged between 65–80 years, and RR > 10.6 for people aged ≥85 years [29,30]. In our study with vaccinated individuals, age ≥ 85 years was the strongest predictor for mortality, but only the predictor with 12^th^ highest weight for hospitalisation. Similarly, we did not find age between 60–80 years to be a strong predictor. In unvaccinated individuals, mortality increases with comorbidities, which is comparable to our results (e.g., CMMS). Likewise, heart, respiratory, and kidney diseases were reported amongst the main predictors in our analyses, as in unvaccinated individuals [31].

A greater than 90-days gap between the first and second dose was positively associated with the three severe COVID-19 outcomes studied. Individuals who received their second dose earlier in 2021 – during the first and second quarter of 2021 – were more likely to be sub-responders. This could have multiple confounders, including the prevalence of less virulent variants in the latter parts of 2021, vaccine waning with time, or prioritisation of high-risk groups for early vaccination. Since the cut-off date of analysis was December 2021, people who were vaccinated earlier in the year would be expected to exhibit more waning. Similarly, time between vaccinations in the UK was defined as 11–12 weeks at the start of the vaccination campaign; later, in May 2021, it was reduced to 8–9 weeks. Therefore, individuals prioritized for earlier vaccination may have been older and with more comorbidities, and would have had a longer follow-up, more time for waning, and an overall higher risk of presenting the study outcomes [2,32,33].

In the sensitivity analysis comparing three different ML methods (XGBoost, LR and DNN), cardiovascular multimorbidity was consistently amongst the main predictors, including chronic heart, respiratory and kidney diseases. Obesity, immunosuppression, diabetes and vaccination in the first quarter of 2021 were also consistently reported across all study outcomes in the three models. All three ML models reported age ≥ 85 years as highly predictive for mortality and moderately predictive for hospitalisation. However, for ICU admission as an outcome, age ≥ 85 years was reported as not predictive by the XGBoost and DNN models, and protective by the LR model (S5 in S1 File). This finding can reflect on how age impacted on the pathways of care at the time of the COVID-19 pandemic. Further qualitative research can investigate potential associated factors, such as the ICU referral criteria or resource allocation considerations at the time of the pandemic [34].

#### Interpretations of the clinical predictive modelling.

Increased mortality in suboptimal vaccination response cases in older people, immunosuppressed groups and people with chronic respiratory disease are in keeping with other studies. A recent prospective study showed that people with chronic obstructive pulmonary disease (COPD), living in care homes and who were having grade B chemotherapy had a significantly higher risk of mortality [35,36]. Conversely, younger people and with a record of flu vaccination in the current year are associated with lower risk of mortality. For example, a recent systematic review and meta-analysis showed that flu vaccination was associated with lower SARS-CoV-2 infection by 24%, death by 32%, hospitalisation by 25% and ICU admissions by 29% [37]. Whilst not establishing a causal relationship, it replicates our findings, suggesting further research could investigate potential confounders in the association between flu vaccination and severe COVID-19 outcomes. Lastly, immunosuppressive treatments and kidney disease are associated to low antibody and cell-mediated immune response, which could be plausibly associated to breakthrough infections after vaccination [38,39].

There are multiple risk factors for hospitalisation due to COVID-19 in vaccinated individuals. Between January and December 2021, there was significant mutation in the SARS-CoV-2 virus (54). The response to vaccination can be disparate in different ethnicities [40]. In addition to chronic respiratory and kidney disease, our study found severe obesity is a strong predictor of sub-optimal response following COVID-19 vaccination [41]. Severely obese people with COVID-19 infection are at a higher risk of ICU admission due to poor respiratory reserve, metabolic derangement [42] and non-alcoholic hepato-steatosis (NASH) [43], low level vasculitis, a prothrombotic and proatherogenic milieu [44,45]. The cytokine storm associated with COVID-19 infection is much more pronounced in severely obese people [46,47]. The immune response after vaccination in severely obese people can be compromised [48], resulting more severe infection causing multi-organ failure, requiring intensive care to provide respiratory, cardiac and renal support. People with severe obesity are more likely to need convalescent plasma or hyperimmune immunoglobulin [49] and therefore ICU admission rate was higher in this group of people.

In our study, we found the risk of mortality, hospitalisation, and ICU admissions in current, ex- and non-smokers were lower, which is counterintuitive. Some studies reported that smoking was a protective factor for COVID-19 outcome [50], while other studies found it increased the risk of mortality [51,52]. Smokers with pre-existing COPD were particularly at a higher risk of the study outcome following SARS-CoV-2 infection [53], even in younger smokers [54]. However, the impact of smoking on the severity of COVID-19 infection can be varied due to its effect on nicotinic receptor and immune system. SARS-CoV-2 virus adheres to ACE-2 receptor which upregulates the expression of ACE 2 receptor in smokers causing more severe disease [55]. On the other hand, chronic smokers may have a dampened immune response, and therefore, may not be able induce a cytokine storm [56,57]. Further study is needed to explain the impact of smoking on COVID-19 related mortality, hospitalisation, and ICU admissions, as our retrospective study cannot make a causal relationship between smoking and COVID-19 outcomes due to the coding of and misclassification of smoking status as potential limitations.

#### Implications of ML modelling on clinical practice.

The sensitivity analysis showed comparable AUROC performance between all three predictive models, with XG-Boost reporting only slightly better results than LR and DNN. This is consistent to other studies comparing ML methodologies [14,58]. This suggests XG-Boost is an accurate predictor of suboptimal COVID-19 vaccine response in high-risk patients, defined as a severe SARS-CoV-2 infection despite being fully vaccinated with two doses. When combined with SHAP explanations, it fulfils the best practice of enabling stakeholders to understand why a particular prediction was reached [59].

While conventional statistical regression methods allow for adjustment for known confounders, with a degree of causality [59], explainable AI emphasises on clinical predictive value. Although predictive models do not imply causal inference, they may be of additional utility for public health interventions by individually flagging high-risk patients through large-scale EHR data. It has been discussed [60] that ML methods are better suited to integrate diverse and complex data sources, such as EHRs. ML also allows combining different types of modelling, and may improve the performance for temporal modelling when using epidemiological data, which may be irregularly sampled.

Improving the content and quality of large-scale datasets, for example, through multimodal linkage at the national or international levels, may improve the predictive performance of explainable AI methods [11,60]. Further research can investigate how to implement feedback mechanisms from centralised data models to clinicians and decision makers. This would enable an action point for complex clinical predictive models based on multimodal linked datasets, which for the moment can only be implemented by centralised data aggregators, rather than as a clinical on-site tool. Thus, predictive models built on primary care data could help identify individuals at high-risk of severe COVID-19 outcomes, or other infectious respiratory diseases, to be prioritised for vaccination booster. In such case, real-world data modelling can inform community-based interventions to promote COVID-19 vaccine uptake in those population subgroups identified as having higher vaccine hesitancy [61].

### Strengths and limitations

Generalisability is a strength of the study as the cohorts were derived from a national population included in ORCHID, the sentinel network of volunteer primary care practitioners who have been recording data on respiratory diseases and vaccinations for over 50 years [62]. To improve representativeness, larger recruitment of practices in the eastern region, and target recruitment into the practice network is necessary [20]. Although the AZD1222 vaccine was our focus, further research can explore how the findings would apply to other COVID-19 vaccines.

This analysis did not actively consider the dynamics of vaccine waning. The analysis was restricted to people who received their second vaccine dose in 2021. As a result, an individual who received a vaccine dose in January has more time to exhibit vaccine waning than someone who got vaccinated in the later months of 2021. Further research may investigate the potential effect of waning on predictions in individuals to whom early vaccination was indicated due to the presence of risk factors, including older age and comorbidities.

Records with missing data were excluded from the cohort, which may potentially introduce bias towards those with high comorbidities. Missing data could signify lower health literacy or lack of visits to primary care, representing potentially better health or lower co-morbidities. However, excluding missing data prevented the introduction of noise into the ML models. Also, since sub-optimal response is associated with higher morbidity and lower health, the direction of the results presented is not expected to be impacted. The results show no evidence of data not being missing at random, which supports the representativeness of the study population.

Although the k-fold cross validation method used is a common method in ML, it may have limitations in the validation results, compared to temporal or external validation. However, an objective of the study was to develop a clinical predictive model from EHR data, which allows for further research to externally validate it across other national or large-scale routine databases.

A limitation from the use of routinely collected data is the possible impact of missing or incorrect vaccination data. For example, data from individuals in the ORCHID database that could not be linked to their vaccination records, or those individuals who were vaccinated outside England. We do not have information about the success of the linkage of data from the ORCHID database with COVID-19 vaccination records. However, pre-specified definitions for exposure and severe COVID-19 outcomes were defined using curated ORCHID variable definitions and ICD10 codes (e.g., hospital admissions). Death certification was linked to the cohort so that primary reason for death could reliably be established.

## Conclusion

Obesity, chronic heart, respiratory and kidney diseases were reported as main predictors of severe COVID-19 in previously vaccinated individuals across ML models, which is comparable to the scientific literature, validating the explainable AI approach. Although age ≥ 85 years was highly predictive for mortality and moderately for hospitalisation, it was negatively associated with ICU admission.

XGBoost can accurately predict occurrence of severe COVID-19 in individuals who have received a two-dose primary series of AZD1222 vaccine. While conventional statistical methods, such as regression analysis, allow for adjustment for known confounders, explainable AI adds an emphasis on clinical predictive value, which may provide a potentially useful combination for targeting public health interventions.

## Supporting information

S1 File S1. Comorbidities based on the (COVID-19) green book Chapter 14a definitions. S2. Cambridge Multimorbidity Score. S3. Algorithm defining COVID-19 vaccination. S4. Results for the sensitivity analysis comparing XGBoost Logistic Regression and Deep Neuronal Neworks. S5. Sensitivity analysis for the Logistic regression model. S6. Sensitivity analysis with Deep Neural Networks using gradients. S7. Tables with the coefficients of the logistic regression trained for predicting the breakthrough cases leading to mortality. S8. Tables with the coefficients of the logistic regression trained for predicting the breakthrough cases leading to hospitalisation. S9. Tables with the coefficients of the logistic regression trained for predicting the breakthrough cases leading to ICU admission. S10. Tables with the SHAP values highlighting the relevance of different input variables in XGBoost trained for predicting breakthrough cases resulting in mortality. S11. Tables with the SHAP values highlighting the relevance of different input variables in XGBoost trained for predicting breakthrough cases resulting in hospitalisation. S12. Tables with the SHAP values obtained from XGBoost trained for the ICU admission prediction.
(ZIP)

## References

[1] Advice on priority groups for COVID-19 vaccination. Joint Committee on Vaccination and Immunisation. 2020. [Online]. Available from: https://www.gov.uk/government/publications/priority-groups-for-coronavirus-covid-19-vaccination-advice-from-the-jcvi-30-december-2020/joint-committee-on-vaccination-and-immunisation-advice-on-priority-groups-for-covid-19-vaccination-30-december-2020

[2] Andrews N, Tessier E, Stowe J, Gower C, Kirsebom F, Simmons R, et al. Duration of Protection against Mild and Severe Disease by Covid-19 Vaccines. N Engl J Med. 2022;386(4):340–50. doi: 10.1056/NEJMoa2115481 35021002 PMC8781262

[3] Katikireddi SV, Cerqueira-Silva T, Vasileiou E, Robertson C, Amele S, Pan J, et al. Two-dose ChAdOx1 nCoV-19 vaccine protection against COVID-19 hospital admissions and deaths over time: a retrospective, population-based cohort study in Scotland and Brazil. Lancet. 2022;399(10319):25–35. doi: 10.1016/S0140-6736(21)02754-9 34942103 PMC8687670

[4] Menni C, May A, Polidori L, Louca P, Wolf J, Capdevila J, et al. COVID-19 vaccine waning and effectiveness and side-effects of boosters: a prospective community study from the ZOE COVID Study. Lancet Infect Dis. 2022;22(7):1002–10. doi: 10.1016/S1473-3099(22)00146-3 35405090 PMC8993156

[5] Skowronski DM, Febriani Y, Ouakki M, Setayeshgar S, El Adam S, Zou M, et al. Two-Dose Severe Acute Respiratory Syndrome Coronavirus 2 Vaccine Effectiveness With Mixed Schedules and Extended Dosing Intervals: Test-Negative Design Studies From British Columbia and Quebec, Canada. Clin Infect Dis. 2022;75(11):1980–92. doi: 10.1093/cid/ciac290 35438175 PMC9047203

[6] Mahase E. Covid-19 booster vaccines: What we know and who’s doing what. BMJ. 2021;374:n2082. doi: 10.1136/bmj.n2082 34417167

[7] Ferri C, Gragnani L, Raimondo V, Visentini M, Giuggioli D, Lorini S, et al. Absent or suboptimal response to booster dose of COVID-19 vaccine in patients with autoimmune systemic diseases. J Autoimmun. 2022;131:102866. doi: 10.1016/j.jaut.2022.102866 35841684 PMC9271490

[8] Agha ME, Blake M, Chilleo C, Wells A, Haidar G. Suboptimal Response to Coronavirus Disease 2019 Messenger RNA Vaccines in Patients With Hematologic Malignancies: A Need for Vigilance in the Postmasking Era. Open Forum Infect Dis. 2021;8(7):ofab353. doi: 10.1093/ofid/ofab353 34337100 PMC8320282

[9] Koff A, Malinis M. Suboptimal Antispike Antibody Levels Following Vaccination in Recipients of Solid Organ Transplant-Variance of Concern. JAMA Netw Open. 2022;5(4):e226880. doi: 10.1001/jamanetworkopen.2022.6880 35412630

[10] Stock PG, Henrich TJ, Segev DL, Werbel WA. Interpreting and addressing suboptimal immune responses after COVID-19 vaccination in solid-organ transplant recipients. J Clin Invest. 2021;131(14):e151178. doi: 10.1172/JCI151178 34143755 PMC8279579

[11] Shah P, Kendall F, Khozin S, Goosen R, Hu J, Laramie J, et al. Artificial intelligence and machine learning in clinical development: a translational perspective. NPJ Digit Med. 2019;2:69. doi: 10.1038/s41746-019-0148-3 31372505 PMC6659652

[12] Abbaspour S, Robbins GK, Blumenthal KG, Hashimoto D, Hopcia K, Mukerji SS, et al. Identifying Modifiable Predictors of COVID-19 Vaccine Side Effects: A Machine Learning Approach. Vaccines (Basel). 2022;10(10):1747. doi: 10.3390/vaccines10101747 36298612 PMC9608090

[13] Bowler S, Papoutsoglou G, Karanikas A, Tsamardinos I, Corley MJ, Ndhlovu LC. A machine learning approach utilizing DNA methylation as an accurate classifier of COVID-19 disease severity. Sci Rep. 2022;12(1):17480. doi: 10.1038/s41598-022-22201-4 36261477 PMC9580434

[14] Ramón A, Torres AM, Milara J, Cascón J, Blasco P, Mateo J. eXtreme Gradient Boosting-based method to classify patients with COVID-19. J Investig Med. 2022;70(7):1472–80. doi: 10.1136/jim-2021-002278 35850970

[15] Morciano M, Stokes J, Kontopantelis E, Hall I, Turner AJ. Excess mortality for care home residents during the first 23 weeks of the COVID-19 pandemic in England: a national cohort study. BMC Med. 2021;19(1):71. doi: 10.1186/s12916-021-01945-2 33663498 PMC7932761

[16] Levin AT, Jylhävä J, Religa D, Shallcross L. COVID-19 prevalence and mortality in longer-term care facilities. Eur J Epidemiol. 2022;37(3):227–34. doi: 10.1007/s10654-022-00861-w 35397704 PMC8994824

[17] Dagan N, Barda N, Kepten E, Miron O, Perchik S, Katz MA, et al. BNT162b2 mRNA Covid-19 Vaccine in a Nationwide Mass Vaccination Setting. N Engl J Med. 2021;384(15):1412–23. doi: 10.1056/NEJMoa2101765 33626250 PMC7944975

[18] NHS Digital. General Practice Data for Planning and Research (GPDPR). [cited 2022 Nov 14]. [Online]. Available from: https://digital.nhs.uk/data-and-information/data-collections-and-data-sets/data-collections/general-practice-data-for-planning-and-research

[19] Wood A, Denholm R, Hollings S, Cooper J, Ip S, Walker V, et al. Linked electronic health records for research on a nationwide cohort of more than 54 million people in England: data resource. BMJ. 2021;373:n826. doi: 10.1136/bmj.n826 33827854 PMC8413899

[20] Leston M, et al. Representativeness, vaccination uptake and COVID clinical outcomes 2020-21 in the UK’s Oxford-RCGP Research and Surveillance Network: cohort profile. JMIR Preprints. 2022;39141. 10.2196/39141 PMC9770023 36534462

[21] COVID-19 - SARS-CoV-2. [Online]. Available from: https://assets.publishing.service.gov.uk/government/uploads/system/uploads/attachment_data/file/1102459/Greenbook-chapter-14a-4September22.pdf

[22] Payne RA, Mendonca SC, Elliott MN, Saunders CL, Edwards DA, Marshall M, et al. Development and validation of the Cambridge Multimorbidity Score. CMAJ. 2020;192(5):E107–14. doi: 10.1503/cmaj.190757 32015079 PMC7004217

[23] Clegg A, Bates C, Young J, Ryan R, Nichols L, Ann Teale E, et al. Development and validation of an electronic frailty index using routine primary care electronic health record data. Age Ageing. 2016;45(3):353–60. doi: 10.1093/ageing/afw039 26944937 PMC4846793

[24] Wong T-T, Yeh P-Y. Reliable Accuracy Estimates from k-Fold Cross Validation. IEEE Trans Knowl Data Eng. 2020;32(8):1586–94. doi: 10.1109/tkde.2019.2912815

[25] Rproject. R: A language and environment for statistical computing. [Online]. Available from: https://www.R-project.org/

[26] de Lusignan S, Jones N, Dorward J, Byford R, Liyanage H, Briggs J, et al. The Oxford Royal College of General Practitioners Clinical Informatics Digital Hub: Protocol to Develop Extended COVID-19 Surveillance and Trial Platforms. JMIR Public Health Surveill. 2020;6(3):e19773. doi: 10.2196/19773 32484782 PMC7333793

[27] de Lusignan S, Liyanage H, McGagh D, Jani BD, Bauwens J, Byford R, et al. COVID-19 Surveillance in a Primary Care Sentinel Network: In-Pandemic Development of an Application Ontology. JMIR Public Health Surveill. 2020;6(4):e21434. doi: 10.2196/21434 33112762 PMC7674143

[28] NHS Digital. Data Security and Protection Toolkit.

[29] Pennington AF, Kompaniyets L, Summers AD, Danielson ML, Goodman AB, Chevinsky JR, et al. Risk of Clinical Severity by Age and Race/Ethnicity Among Adults Hospitalized for COVID-19-United States, March-September 2020. Open Forum Infect Dis. 2020;8(2):ofaa638. doi: 10.1093/ofid/ofaa638 33553477 PMC7798738

[30] Booth A, Reed AB, Ponzo S, Yassaee A, Aral M, Plans D, et al. Population risk factors for severe disease and mortality in COVID-19: A global systematic review and meta-analysis. PLoS One. 2021;16(3):e0247461. doi: 10.1371/journal.pone.0247461 33661992 PMC7932512

[31] Kompaniyets L, Pennington AF, Goodman AB, Rosenblum HG, Belay B, Ko JY, et al. Underlying Medical Conditions and Severe Illness Among 540,667 Adults Hospitalized With COVID-19, March 2020-March 2021. Prev Chronic Dis. 2021;18:E66. doi: 10.5888/pcd18.210123 34197283 PMC8269743

[32] NHS England. COVID-19 vaccination programme: FAQs on second doses. [Online]. Available from: https://www.england.nhs.uk/coronavirus/documents/covid-19-vaccination-programme-faqs-on-second-doses/#:~:text=Clinics should schedule second dose,unless there are exceptional circumstances.

[33] Haq MA, Roy AK, Ahmed R, Kuddusi RU, Sinha M, Hossain MS, et al. Antibody longevity and waning following COVID-19 vaccination in a 1-year longitudinal cohort in Bangladesh. Sci Rep. 2024;14(1):11467. doi: 10.1038/s41598-024-61922-6 38769324 PMC11106241

[34] Emanuel EJ, Persad G, Upshur R, Thome B, Parker M, Glickman A, et al. Fair Allocation of Scarce Medical Resources in the Time of Covid-19. N Engl J Med. 2020;382(21):2049–55. doi: 10.1056/NEJMsb2005114 32202722

[35] “Vaccinations in United Kingdom.” [Online]. Available from: https://coronavirus.data.gov.uk/details/vaccinations

[36] Hirsh J, Htay T, Bhalla S, Nguyen V, Cervantes J. Breakthrough SARS-CoV-2 infections after COVID-19 immunization. J Investig Med. 2022;70(6):1429–32. doi: 10.1136/jim-2021-002131 35768140

[37] Zeynali Bujani M, Behnampour M, Rahimi N, Safari T, Khazaei Feizabad A, Hossein Sarbazi A, et al. The Effect of Influenza Vaccination on COVID-19 Morbidity, Severity and Mortality: Systematic Review and Meta-Analysis. Malays J Med Sci. 2021;28(6):20–31. doi: 10.21315/mjms2021.28.6.3 35002487 PMC8715887

[38] Boongird S, Chuengsaman P, Setthaudom C, Nongnuch A, Assanatham M, Phanprasert S, et al. Short-Term Immunogenicity Profiles and Predictors for Suboptimal Immune Responses in Patients with End-Stage Kidney Disease Immunized with Inactivated SARS-CoV-2 Vaccine. Infect Dis Ther. 2022;11(1):351–65. doi: 10.1007/s40121-021-00574-9 34859359 PMC8639296

[39] Whelan MG, Santacroce L, Masto L, Qian G, Kowalski E, Vanni K, et al. Predictors of low spike antibody response in patients with systemic rheumatic disease after an initial course of COVID-19 vaccination. Clin Rheumatol. 2023;42(6):1695–700. doi: 10.1007/s10067-023-06512-z 36656454 PMC9850319

[40] Lv G, Yuan J, Xiong X, Li M. Mortality Rate and Characteristics of Deaths Following COVID-19 Vaccination. Front Med (Lausanne). 2021;8:670370. doi: 10.3389/fmed.2021.670370 34055843 PMC8160119

[41] Manz KM, Schwettmann L, Mansmann U, Maier W. Area Deprivation and COVID-19 Incidence and Mortality in Bavaria, Germany: A Bayesian Geographical Analysis. Front Public Health. 2022;10:927658. doi: 10.3389/fpubh.2022.927658 35910894 PMC9334899

[42] Mathur R, Rentsch CT, Morton CE, Hulme WJ, Schultze A, MacKenna B, et al. Ethnic differences in SARS-CoV-2 infection and COVID-19-related hospitalisation, intensive care unit admission, and death in 17 million adults in England: an observational cohort study using the OpenSAFELY platform. Lancet. 2021;397(10286):1711–24. doi: 10.1016/S0140-6736(21)00634-6 33939953 PMC8087292

[43] Chudasama YV, Zaccardi F, Gillies CL, Razieh C, Yates T, Kloecker DE, et al. Patterns of multimorbidity and risk of severe SARS-CoV-2 infection: an observational study in the U.K. BMC Infect Dis. 2021;21(1):908. doi: 10.1186/s12879-021-06600-y 34481456 PMC8418288

[44] Kaur N, Singh R, Dar Z, Bijarnia RK, Dhingra N, Kaur T. Genetic comparison among various coronavirus strains for the identification of potential vaccine targets of SARS-CoV2. Infect Genet Evol. 2021;89:104490. doi: 10.1016/j.meegid.2020.104490 32745811 PMC7395230

[45] Bose T, Pant N, Pinna NK, Bhar S, Dutta A, Mande SS. Does immune recognition of SARS-CoV2 epitopes vary between different ethnic groups? Virus Res. 2021;305:198579. doi: 10.1016/j.virusres.2021.198579 34560183 PMC8453877

[46] Michalakis K, Ilias I. SARS-CoV-2 infection and obesity: Common inflammatory and metabolic aspects. Diabetes Metab Syndr. 2020;14(4):469–71. doi: 10.1016/j.dsx.2020.04.033 32387864 PMC7189186

[47] Sales-Peres SH de C, de Azevedo-Silva LJ, Bonato RCS, Sales-Peres M de C, Pinto AC da S, Santiago Junior JF. Coronavirus (SARS-CoV-2) and the risk of obesity for critically illness and ICU admitted: Meta-analysis of the epidemiological evidence. Obes Res Clin Pract. 2020;14(5):389–97. doi: 10.1016/j.orcp.2020.07.007 32773297 PMC7396969

[48] Rebello CJ, Kirwan JP, Greenway FL. Obesity, the most common comorbidity in SARS-CoV-2: is leptin the link?. Int J Obes (Lond). 2020;44(9):1810–7. doi: 10.1038/s41366-020-0640-5 32647360 PMC7347260

[49] Magdy Beshbishy A, Hetta HF, Hussein DE, Saati AA, C Uba C, Rivero-Perez N, et al. Factors Associated with Increased Morbidity and Mortality of Obese and Overweight COVID-19 Patients. Biology (Basel). 2020;9(9):280. doi: 10.3390/biology9090280 32916925 PMC7564335

[50] Westheim AJF, Bitorina AV, Theys J, Shiri-Sverdlov R. COVID-19 infection, progression, and vaccination: Focus on obesity and related metabolic disturbances. Obes Rev. 2021;22(10):e13313. doi: 10.1111/obr.13313 34269511 PMC8420274

[51] Ali FEM, Mohammedsaleh ZM, Ali MM, Ghogar OM. Impact of cytokine storm and systemic inflammation on liver impairment patients infected by SARS-CoV-2: Prospective therapeutic challenges. World J Gastroenterol. 2021;27(15):1531–52. doi: 10.3748/wjg.v27.i15.1531 33958841 PMC8058655

[52] Bhaskar S, Sinha A, Banach M, Mittoo S, Weissert R, Kass JS, et al. Cytokine Storm in COVID-19-Immunopathological Mechanisms, Clinical Considerations, and Therapeutic Approaches: The REPROGRAM Consortium Position Paper. Front Immunol. 2020;11:1648. doi: 10.3389/fimmu.2020.01648 32754159 PMC7365905

[53] Amin MT, Fatema K, Arefin S, Hussain F, Bhowmik DR, Hossain MS. Obesity, a major risk factor for immunity and severe outcomes of COVID-19. Biosci Rep. 2021;41(8):BSR20210979. doi: 10.1042/BSR20210979 34350941 PMC8380923

[54] Piechotta V, Chai KL, Valk SJ, Doree C, Monsef I, Wood EM, et al. Convalescent plasma or hyperimmune immunoglobulin for people with COVID-19: a living systematic review. Cochrane Database Syst Rev. 2020;7(7):CD013600. doi: 10.1002/14651858.CD013600.pub2 32648959 PMC7389743

[55] Rajput DV. Systematic review of the prevalence of current smoking among hospitalized COVID-19 patients in China: could nicotine be a therapeutic option?: Comment. Intern Emerg Med. 2021;16(1):233–4. doi: 10.1007/s11739-020-02396-y 32514683 PMC7279633

[56] Salah HM, Sharma T, Mehta J. Smoking Doubles the Mortality Risk in COVID-19: A Meta-Analysis of Recent Reports and Potential Mechanisms. Cureus. 2020;12(10):e10837. doi: 10.7759/cureus.10837 33173643 PMC7647838

[57] Lippi G, Henry BM. Active smoking is not associated with severity of coronavirus disease 2019 (COVID-19). Eur J Intern Med. 2020;75:107–8. doi: 10.1016/j.ejim.2020.03.014 32192856 PMC7118593

[58] Nwanosike EM, Conway BR, Merchant HA, Hasan SS. Potential applications and performance of machine learning techniques and algorithms in clinical practice: A systematic review. Int J Med Inform. 2022;159:104679. doi: 10.1016/j.ijmedinf.2021.104679 34990939

[59] de Hond AAH, Leeuwenberg AM, Hooft L, Kant IMJ, Nijman SWJ, van Os HJA, et al. Guidelines and quality criteria for artificial intelligence-based prediction models in healthcare: a scoping review. NPJ Digit Med. 2022;5(1):2. doi: 10.1038/s41746-021-00549-7 35013569 PMC8748878

[60] Syrowatka A, Kuznetsova M, Alsubai A, Beckman AL, Bain PA, Craig KJT, et al. Leveraging artificial intelligence for pandemic preparedness and response: a scoping review to identify key use cases. NPJ Digit Med. 2021;4(1):96. doi: 10.1038/s41746-021-00459-8 34112939 PMC8192906

[61] Chaudhry T, Tum P, Tam HZ, Brentnall A, Smethurst H, Kielmann K, et al. COVER-ME: developing and evaluating community-based interventions to promote vaccine uptake for COVID-19 and influenza in East London minority ethnicity (ME) and underserved individuals - protocol for a pilot randomised controlled trial. BMJ Open. 2025;15(3):e092568. doi: 10.1136/bmjopen-2024-092568 40107676 PMC11927436

[62] de Lusignan S, Correa A, Smith GE, Yonova I, Pebody R, Ferreira F, et al. RCGP Research and Surveillance Centre: 50 years’ surveillance of influenza, infections, and respiratory conditions. Br J Gen Pract. 2017;67(663):440–1. doi: 10.3399/bjgp17X692645 28963401 PMC5604796

