# Supplementary material for: Prediction of COVID-19 hospitalisation, ICU admission or death following ChAdOx1 vaccination using artificial intelligence: A clinical predictive model from the English RAVEN study
Source: PLoS One. 2026 Feb 20;21(2):e0336449. doi: 10.1371/journal.pone.0336449 (PMC12923009; doi:10.1371/journal.pone.0336449)
Supplement: S1 File — S1. Comorbidities based on the (COVID-19) green book Chapter 14a definitions. S2. Cambridge Multimorbidity Score. S3. Algorithm defining COVID-19 vaccination. S4. Results for the sensitivity analysis comparing XGBoost Logistic Regression and Deep Neuronal Neworks. S5. Sensitivity analysis for the Logistic regression model. S6. Sensitivity analysis with Deep Neural Networks using gradients. S7. Tables with the coefficients of the logistic regression trained for predicting the breakthrough cases leading to mortality. S8. Tables with the coefficients of the logistic regression trained for predicting the breakthrough cases leading to hospitalisation. S9. Tables with the coefficients of the logistic regression trained for predicting the breakthrough cases leading to ICU admission. S10. Tables with the SHAP values highlighting the relevance of different input variables in XGBoost trained for predicting breakthrough cases resulting in mortality. S11. Tables with the SHAP values highlighting the relevance of different input variables in XGBoost trained for predicting breakthrough cases resulting in hospitalisation. S12. Tables with the SHAP values obtained from XGBoost trained for the ICU admission prediction. (ZIP) [file pone.0336449.s001.zip › S9_RAVEN_AI_20260205.docx]

Supplementary material 9

### S9. Tables with the coefficients of the logistic regression trained for predicting the breakthrough cases leading to ICU admission.

| **Features** | **LR Coefficients** | **Lower CI** | **Upper CI** |
| --- | --- | --- | --- |
| calendar time | -1.627740041 | -1.655300471 | -1.600179611 |
| Active Smoker | -1.611940766 | -1.639501196 | -1.584380336 |
| BMI normal | -1.44383657 | -1.471397 | -1.41627614 |
| CMMS_2 | -1.212378273 | -1.239938703 | -1.184817843 |
| Immunosuppression | 1.210845308 | 1.183284878 | 1.238405738 |
| BMI over weight | -1.149262365 | -1.176822795 | -1.121701935 |
| Ethnicity black | -1.110804401 | -1.138364831 | -1.083243971 |
| Ethnicity white | -1.089556262 | -1.117116692 | -1.061995832 |
| BMI under weight | -1.05373387 | -1.0812943 | -1.02617344 |
| Non-Smoker | -1.016114472 | -1.043674902 | -0.988554042 |
| age 85+ | -0.993870803 | -1.021431233 | -0.966310373 |
| NewNHSRegion South East | -0.927749924 | -0.955310354 | -0.900189494 |
| ChronicKidneyDisease | 0.913095756 | 0.885535326 | 0.940656186 |
| CMMS_1 | -0.910150474 | -0.937710904 | -0.882590044 |
| Sex | -0.880736847 | -0.908297277 | -0.853176417 |
| CMMS_3 | -0.835097811 | -0.862658241 | -0.807537381 |
| IMDQuintile_4 | -0.798099778 | -0.825660208 | -0.770539348 |
| IMDQuintile_5 | -0.783568146 | -0.811128576 | -0.756007716 |
| NewNHSRegion East of England | -0.746580793 | -0.774141223 | -0.719020363 |
| IMDQuintile_3 | -0.697664698 | -0.725225128 | -0.670104268 |
| Ex-Smoker | -0.688982992 | -0.716543422 | -0.661422562 |
| age [35,40) | -0.649237297 | -0.676797727 | -0.621676867 |
| age [16,25) | -0.601628669 | -0.629189099 | -0.574068239 |
| IMDQuintile_2 | -0.586983255 | -0.614543686 | -0.559422825 |
| BMI severe obesity | 0.577934886 | 0.550374456 | 0.605495316 |
| Diabetes | 0.571556148 | 0.543995718 | 0.599116578 |
| Ethnicity other | -0.541016992 | -0.568577422 | -0.513456562 |
| ChronicRespiratoryDisease | 0.538916315 | 0.511355885 | 0.566476745 |
| NewNHSRegion Midlands | -0.463699714 | -0.491260144 | -0.436139284 |
| IMDQuintile_1 | -0.450722353 | -0.478282783 | -0.423161922 |
| NewNHSRegion North East and Yorkshire | -0.405090683 | -0.432651113 | -0.377530253 |
| CMMS_4 | -0.359411671 | -0.386972102 | -0.331851241 |
| age [80,85) | -0.319339304 | -0.346899734 | -0.291778874 |
| age [40,45) | -0.298139694 | -0.325700124 | -0.270579264 |
| Ethnicity asian | -0.297400392 | -0.324960822 | -0.269839962 |
| NewNHSRegion South West | -0.289075821 | -0.316636251 | -0.261515391 |
| Ethnicity mix | -0.278260183 | -0.305820613 | -0.250699753 |
| ChronicHeartDisease | 0.26626746 | 0.23870703 | 0.29382789 |
| NewNHSRegion London | -0.264504819 | -0.292065249 | -0.236944388 |
| BMI obese | -0.24814031 | -0.27570074 | -0.22057988 |
| age [45,50) | -0.243559474 | -0.271119904 | -0.215999044 |
| NewNHSRegion North West | -0.220336476 | -0.247896906 | -0.192776046 |
| age [50,55) | -0.187707454 | -0.215267884 | -0.160147024 |
| age [25,35) | -0.179984054 | -0.207544484 | -0.152423624 |
| age [75,80) | -0.163037469 | -0.190597899 | -0.135477039 |
| SevereMentalIllness | -0.156805052 | -0.184365482 | -0.129244622 |
| Flu Vaccination In Current Year | -0.15668382 | -0.18424425 | -0.12912339 |
| age [55,60) | 0.144635376 | 0.117074946 | 0.172195806 |
| ChronicLiverDisease | 0.124810691 | 0.097250261 | 0.152371121 |
| age [60,65) | 0.121642515 | 0.094082085 | 0.149202945 |
| Asplenia | -0.109816529 | -0.137376959 | -0.082256099 |
| age [70,75) | 0.082367478 | 0.054807048 | 0.109927908 |
| ChronicNeurologicalDisease | 0.043663174 | 0.016102744 | 0.071223605 |
| age [65,70) | -0.029179381 | -0.056739811 | -0.001618951 |
| Time between doses | 0.000221438 | -0.027338992 | 0.027781868 |

BMI – body mass index; CMMS – Cambridge multimorbidity score; IMD – index of multiple deprivation
