# Supplementary material for: Prediction of COVID-19 hospitalisation, ICU admission or death following ChAdOx1 vaccination using artificial intelligence: A clinical predictive model from the English RAVEN study
Source: PLoS One. 2026 Feb 20;21(2):e0336449. doi: 10.1371/journal.pone.0336449 (PMC12923009; doi:10.1371/journal.pone.0336449)

Supplementary material 6

### S6. Sensitivity analysis with Deep Neural Networks using gradients

Deep Neural Networks using gradients: Methods

*Fully connected neural network*

Neural networks are non-linear machine learning models that utilize multiple layers to progressively extract features from the raw input and map these extracted features to the model predictions. The parameters are trained using stochastic gradient descent to minimize the deviation between model outputs and true labels.

In this work, a simple fully connected neural network that has one hidden layer with 64 nodes and an output layer with a single node. The hidden layer is followed by the rectified linear unit activation function, while the output layer is followed by a sigmoid activation function*.* For training this model, we used a batch-size of 1024 examples and Adam optimiser with a learning rate of 0.001. All these parameters and design choices are based on standard practices in deep learning for binary classification/prediction and were found to effective for the current tasks (based on cross-validation performance).

For deep learning models, we use a gradient-based approach to establish the feature relevance of the input features. For an input example, the trained model is used to obtain the prediction, and the gradient of the model input with respect to the model output is computed. The magnitude of the gradient corresponding to a feature in the input represents the impact of this feature on the model output. Larger magnitude corresponds to more relevance. We aggregate these example-specific feature relevance across all examples to obtain a global estimate of feature relevance in the trained DNNs.

Deep Neural Networks using gradients: Results

Overall, the DNN models trained for mortality, hospitalisation and ICU admission prediction show that people with multimorbidity, obesity, old age and immunosuppression were at higher risk of the study outcomes. (see Figure 14 to Figure 16)

Age ≥85 years, low CMMS, age (50-54), calendar time, active smoker status and black ethnicity were variables associated to higher mortality in the DNN trained model.

Figure 14 - Top 25 relevant features as per average absolute magnitude of gradients in DNN trained for mortality prediction.


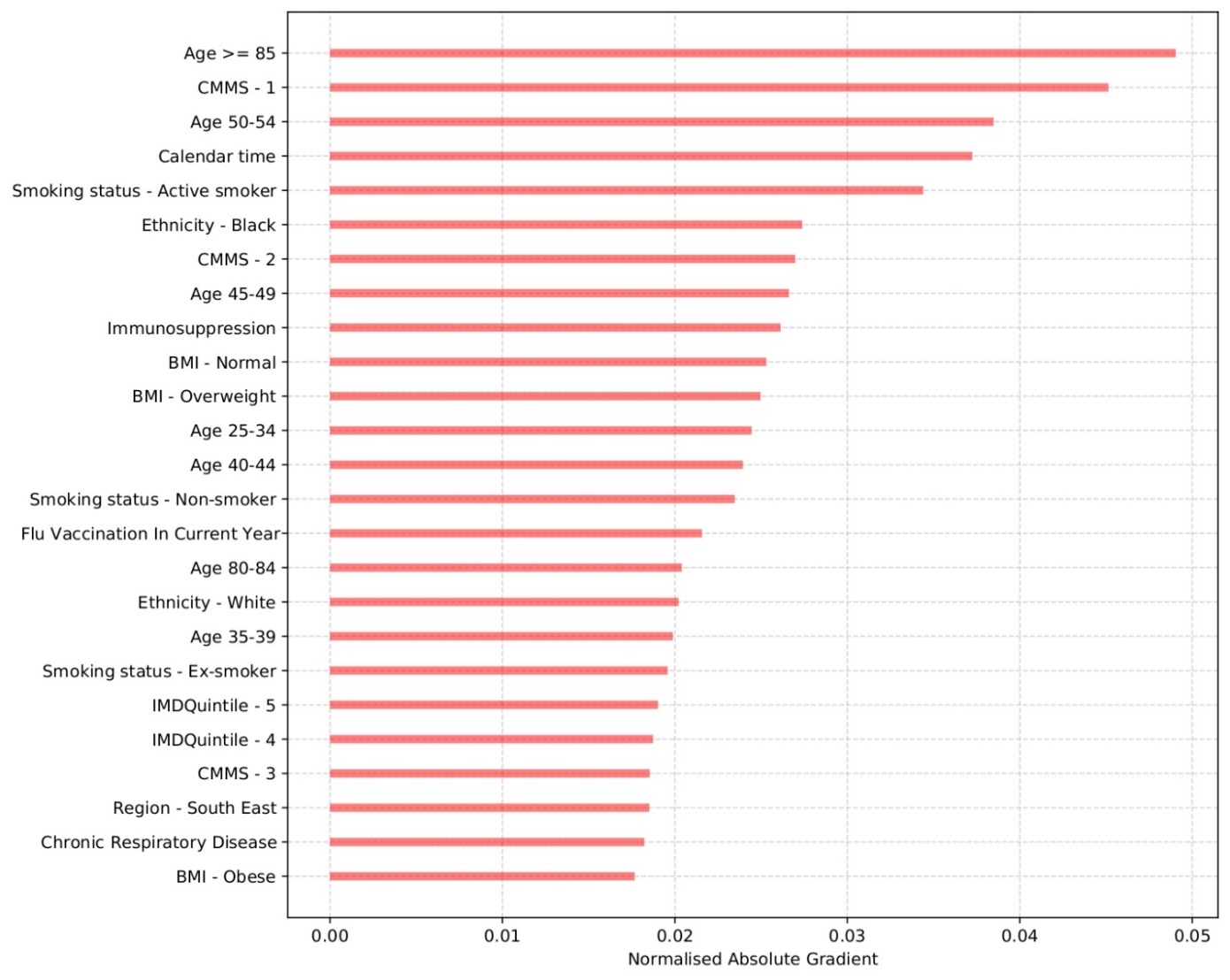


For the prediction of hospitalisation with the DNN trained model, active smoker status, date of vaccination (calendar time), normal BMI, and immunosuppression reported the highest associations to the outcome.

Figure 15 - Top relevant features as per average absolute magnitude of gradients in DNN trained for hospitalisation prediction.


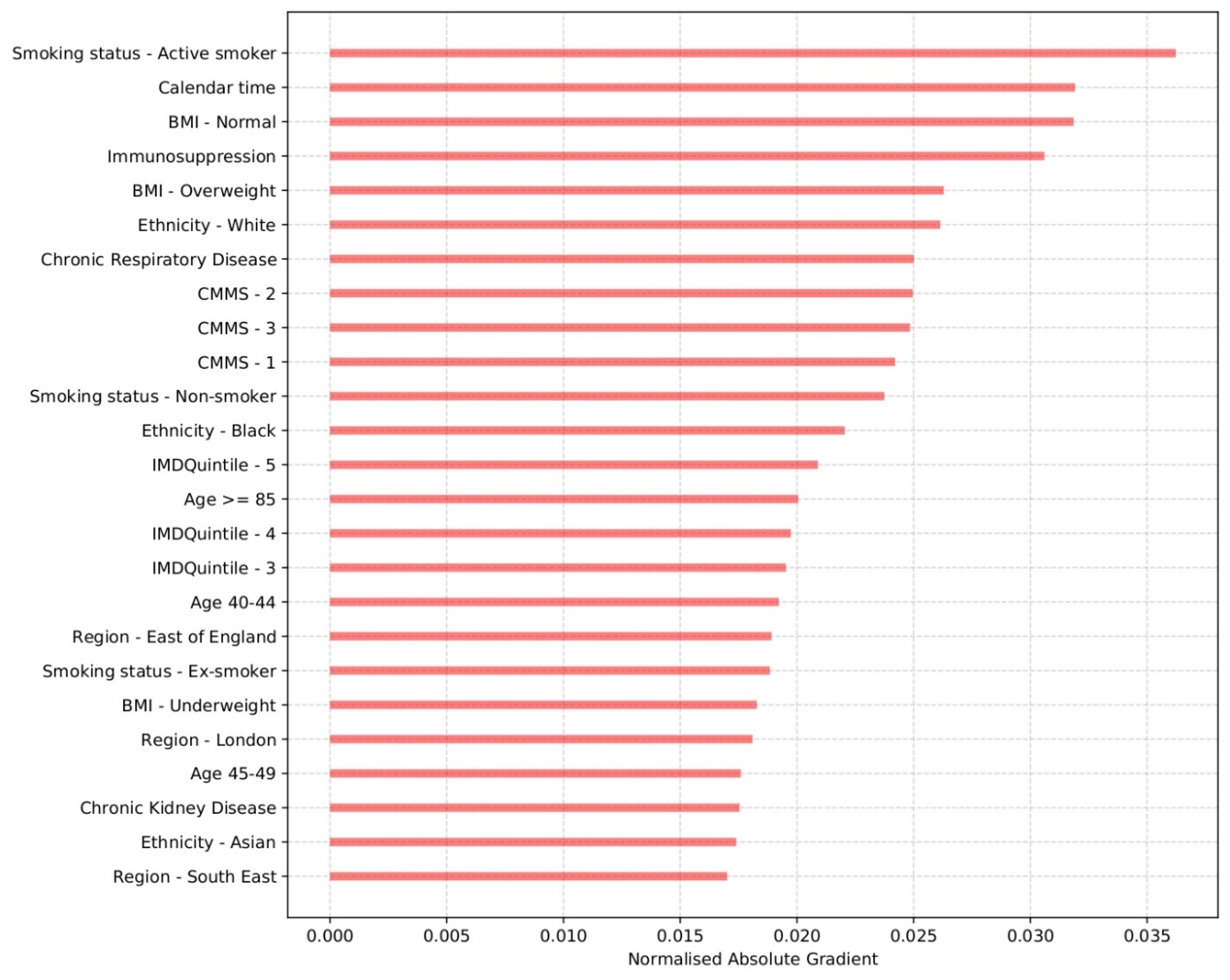


For the prediction of ICU admission with the DNN trained model, normal BMI, calendar time, active smoker status, and immunosuppression reported the highest associations to the outcome.

Figure 16 - Top relevant features as per average absolute magnitude of gradients in DNN trained for ICU admission prediction.


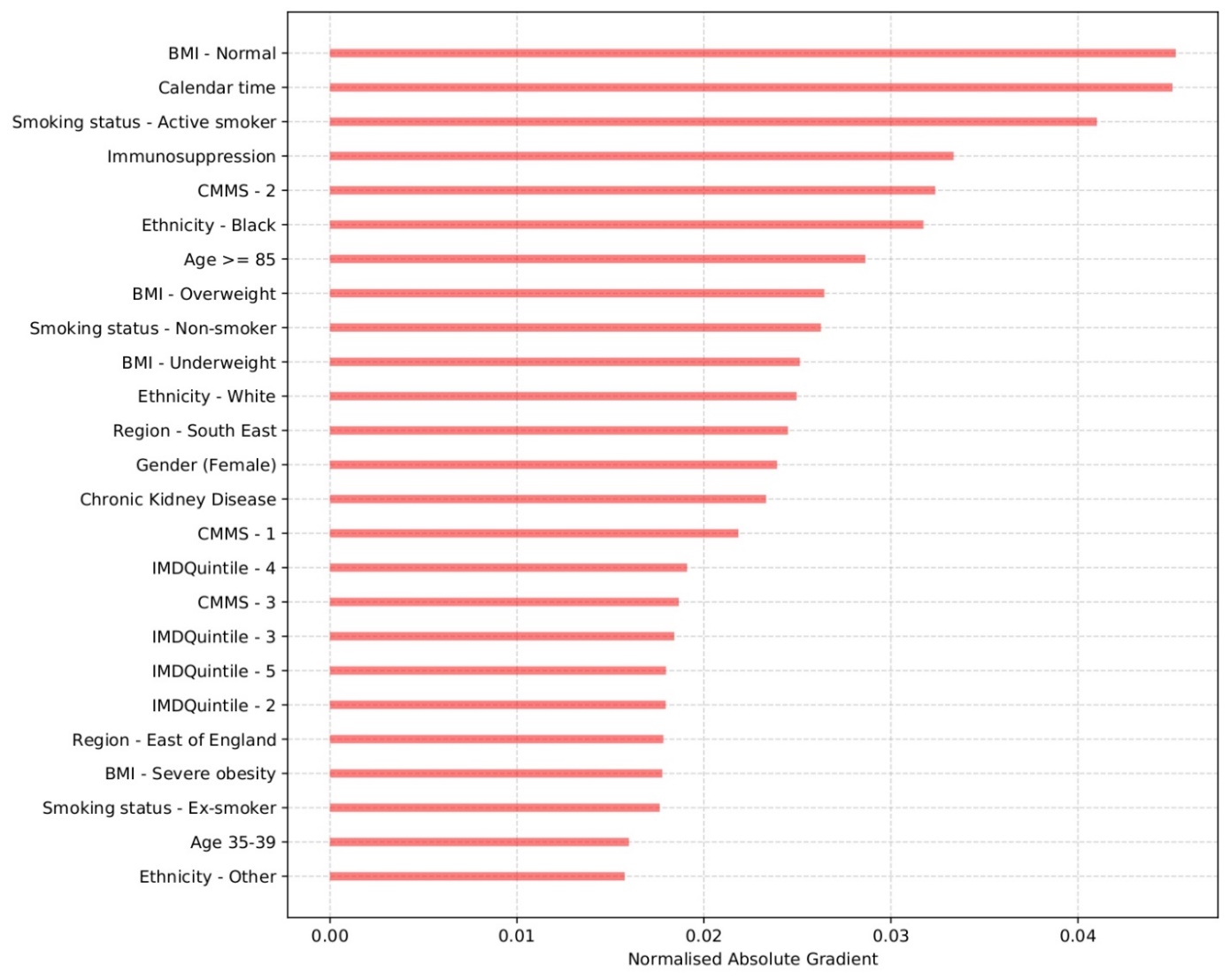

Supplement: S1 File — S1. Comorbidities based on the (COVID-19) green book Chapter 14a definitions. S2. Cambridge Multimorbidity Score. S3. Algorithm defining COVID-19 vaccination. S4. Results for the sensitivity analysis comparing XGBoost Logistic Regression and Deep Neuronal Neworks. S5. Sensitivity analysis for the Logistic regression model. S6. Sensitivity analysis with Deep Neural Networks using gradients. S7. Tables with the coefficients of the logistic regression trained for predicting the breakthrough cases leading to mortality. S8. Tables with the coefficients of the logistic regression trained for predicting the breakthrough cases leading to hospitalisation. S9. Tables with the coefficients of the logistic regression trained for predicting the breakthrough cases leading to ICU admission. S10. Tables with the SHAP values highlighting the relevance of different input variables in XGBoost trained for predicting breakthrough cases resulting in mortality. S11. Tables with the SHAP values highlighting the relevance of different input variables in XGBoost trained for predicting breakthrough cases resulting in hospitalisation. S12. Tables with the SHAP values obtained from XGBoost trained for the ICU admission prediction. (ZIP) [file pone.0336449.s001.zip › S6_RAVEN_AI_20260205.docx]
