# Supplementary material for: Prediction of COVID-19 hospitalisation, ICU admission or death following ChAdOx1 vaccination using artificial intelligence: A clinical predictive model from the English RAVEN study
Source: PLoS One. 2026 Feb 20;21(2):e0336449. doi: 10.1371/journal.pone.0336449 (PMC12923009; doi:10.1371/journal.pone.0336449)

Supplementary material 5

### S5. Sensitivity analysis for the Logistic regression model

Logistic regression model: Methods

Machine learning (ML) models including logistic regression (LR), extreme gradient boosting trees (XGBoost) and a fully connected neural network (DNN) were trained to predict the suboptimal response cases exhibiting the study outcomes – COVID-19 related mortality, hospitalisation and ICU admissions among individuals with a two-dose primary series of AZD1222 vaccine.

The trained models were used to identify characteristics of the sub-responders resulting in the study outcomes. Then, a post-hoc analysis was performed on the trained models to identify variables that were positively associated with the positive model predictions, which is described below for each model:

*Logistic regression*

We used it to estimate the parameters of a logit model that computes the probability of an event by modelling its log-odds as a linear combination of the independent input variables or predictors. Estimated parameters are the coefficients of a linear combination, and the weight or coefficient calculated for each feature/predictor provides information regarding the association of the predictor with the output. The magnitude of each coefficient in a trained LR model helps determine the influence of each variable on the outcome of interest. The polarity or sign of the coefficient helps in determining the nature of association with the outcome of interest. Negative and positive signs indicate negative and positive associations, respectively.

Logistic regression model: Results

The individuals with age ≥85 (1.86 [1.82,1.89]), immunosuppression (1.34 [1.30,1.37]), age (80-84) (0.98 [0.94,1.01]), and chronic respiratory disease (0.99 [0.96,1.03])were associated with higher risk of suboptimal response cases leading to COVID-19 related mortality in the LR model (see Figure 11 and S1 Appendix 7. Tables with the coefficients of the logistic regression trained for predicting the breakthrough cases leading to mortality). Positive coefficients imply positive association between the variable and the outcome. Calendar time of infection, age 50-54, and active smoker status were found to be negatively associated to the outcome.

Figure 11 - Top 25 most relevant features in Logistic Regression (LR) for mortality prediction as per magnitude of the LR coefficients. For coefficients for all input features see S1 Appendix 7. Tables with the coefficients of the logistic regression trained for predicting the breakthrough cases leading to mortality.


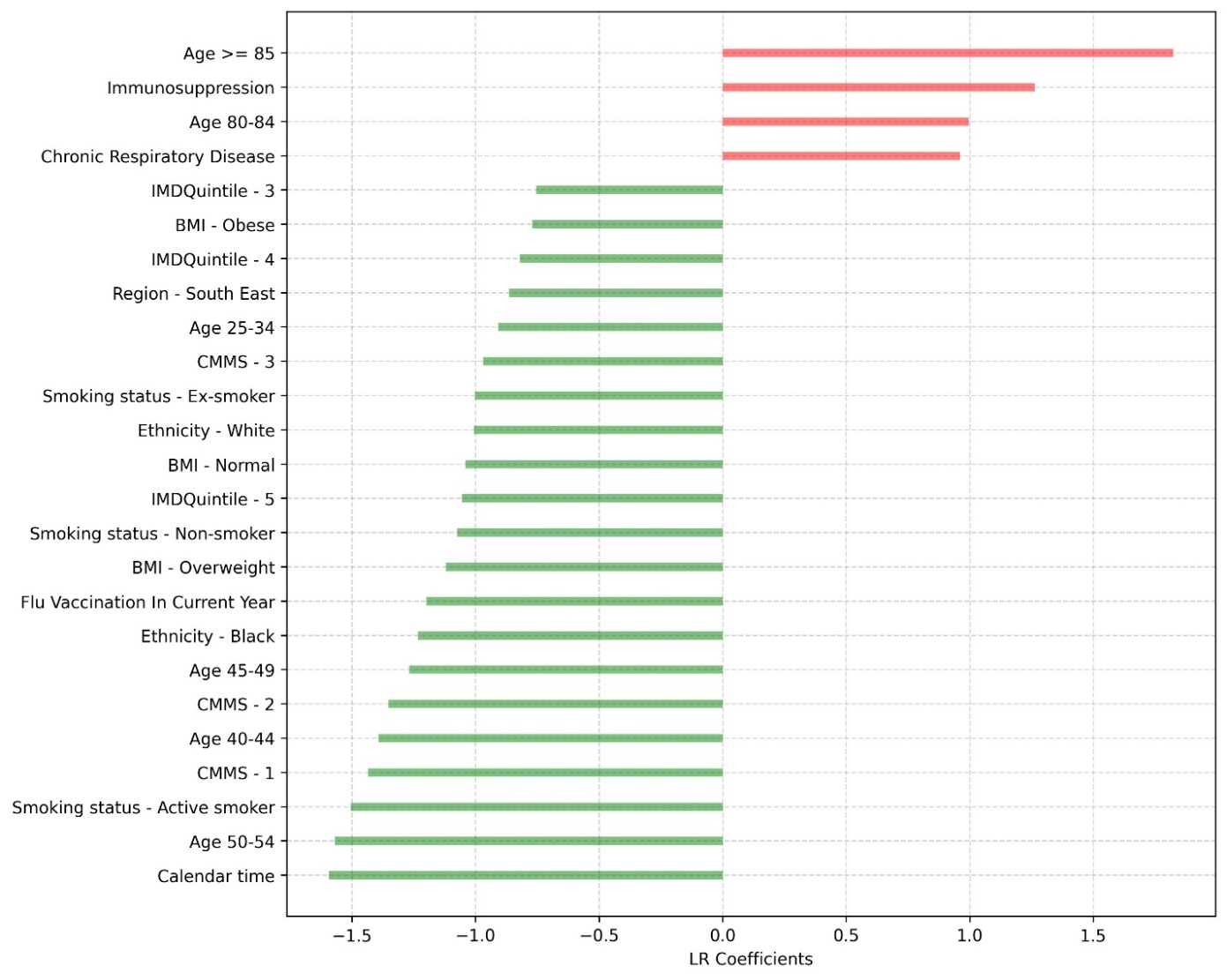


Immunosuppression (0.96 [0.87,1.04]), chronic respiratory disease (0.80 [0.72, 0.89]), age ≥85 years (0.53 [0.45, 0.62]) and chronic kidney disease (0.49 [0.41, 0.58) reported the highest positive associations to suboptimal responses leading to COVID-19 related hospitalisation in the trained LR model (see Figure 12 and S1 Appendix 8. Tables with the coefficients of the logistic regression trained for predicting the breakthrough cases leading to hospitalisation.). Active smoking, calendar time of vaccination, normal BMI and the CMMS groups 1-3 were negatively associated to the outcome.

Figure 12 - Top 25 most relevant features in Logistic Regression (LR) for COVID-19 related hospitalisation prediction as per magnitude of the LR coefficients. For coefficients for all input features see S1 Appendix 8. Tables with the coefficients of the logistic regression trained for predicting the breakthrough cases leading to hospitalisation.


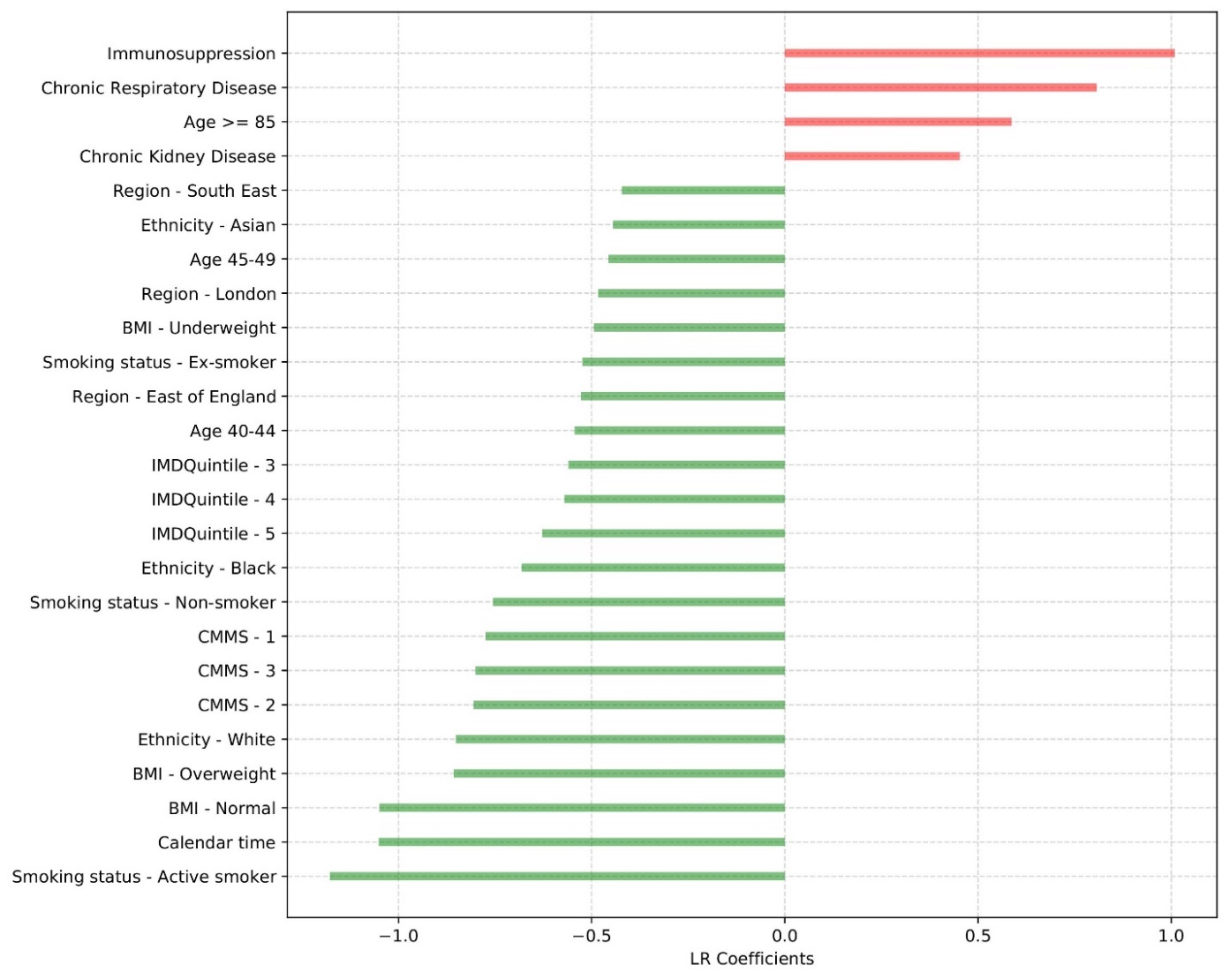


Immunosuppression (1.21 [1.18,1.24]), chronic kidney disease (0.91 [0.89, 0.94), and diabetes (0.57 [0.54, 0.59]), were positively correlated with suboptimal response cases leading to COVID-19 related ICU admission in the trained LR model (see Figure 13 and S1 Appendix 9. Tables with the coefficients of the logistic regression trained for predicting the breakthrough cases leading to ICU admission.). Normal BMI, active smoking and calendar time were negatively associated to the ICU admission.

Figure 13 - Top 25 most relevant features in Logistic Regression (LR) for ICU admission prediction as per magnitude of the LR coefficients. For coefficients for all input features see S1 Appendix 9. Tables with the coefficients of the logistic regression trained for predicting the breakthrough cases leading to ICU admission.


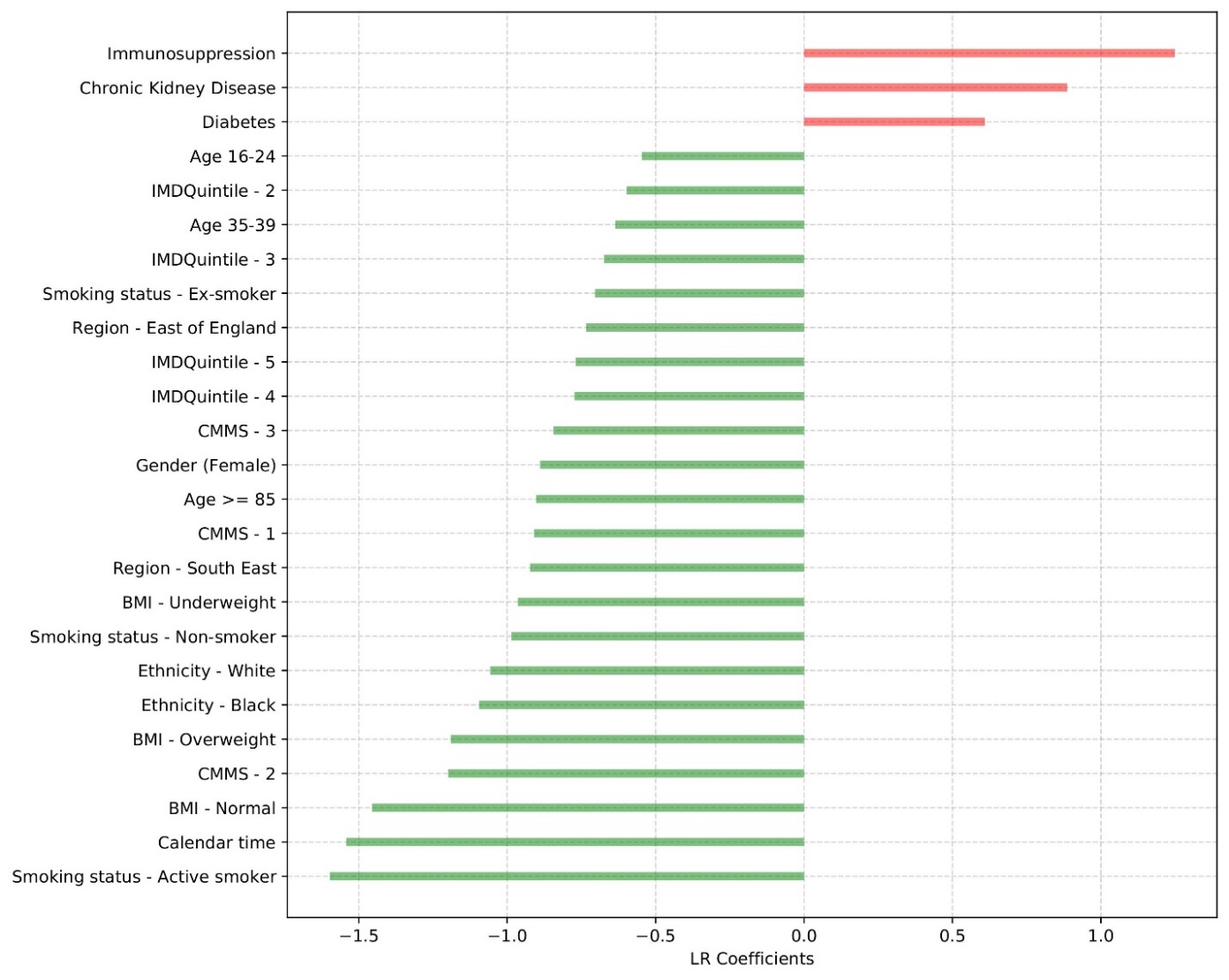

Supplement: S1 File — S1. Comorbidities based on the (COVID-19) green book Chapter 14a definitions. S2. Cambridge Multimorbidity Score. S3. Algorithm defining COVID-19 vaccination. S4. Results for the sensitivity analysis comparing XGBoost Logistic Regression and Deep Neuronal Neworks. S5. Sensitivity analysis for the Logistic regression model. S6. Sensitivity analysis with Deep Neural Networks using gradients. S7. Tables with the coefficients of the logistic regression trained for predicting the breakthrough cases leading to mortality. S8. Tables with the coefficients of the logistic regression trained for predicting the breakthrough cases leading to hospitalisation. S9. Tables with the coefficients of the logistic regression trained for predicting the breakthrough cases leading to ICU admission. S10. Tables with the SHAP values highlighting the relevance of different input variables in XGBoost trained for predicting breakthrough cases resulting in mortality. S11. Tables with the SHAP values highlighting the relevance of different input variables in XGBoost trained for predicting breakthrough cases resulting in hospitalisation. S12. Tables with the SHAP values obtained from XGBoost trained for the ICU admission prediction. (ZIP) [file pone.0336449.s001.zip › S5_RAVEN_AI_20260205.docx]
