# Supplementary material for: Prediction of COVID-19 hospitalisation, ICU admission or death following ChAdOx1 vaccination using artificial intelligence: A clinical predictive model from the English RAVEN study
Source: PLoS One. 2026 Feb 20;21(2):e0336449. doi: 10.1371/journal.pone.0336449 (PMC12923009; doi:10.1371/journal.pone.0336449)
Supplement: S1 File — S1. Comorbidities based on the (COVID-19) green book Chapter 14a definitions. S2. Cambridge Multimorbidity Score. S3. Algorithm defining COVID-19 vaccination. S4. Results for the sensitivity analysis comparing XGBoost Logistic Regression and Deep Neuronal Neworks. S5. Sensitivity analysis for the Logistic regression model. S6. Sensitivity analysis with Deep Neural Networks using gradients. S7. Tables with the coefficients of the logistic regression trained for predicting the breakthrough cases leading to mortality. S8. Tables with the coefficients of the logistic regression trained for predicting the breakthrough cases leading to hospitalisation. S9. Tables with the coefficients of the logistic regression trained for predicting the breakthrough cases leading to ICU admission. S10. Tables with the SHAP values highlighting the relevance of different input variables in XGBoost trained for predicting breakthrough cases resulting in mortality. S11. Tables with the SHAP values highlighting the relevance of different input variables in XGBoost trained for predicting breakthrough cases resulting in hospitalisation. S12. Tables with the SHAP values obtained from XGBoost trained for the ICU admission prediction. (ZIP) [file pone.0336449.s001.zip › S4_RAVEN_AI_20260205.docx]

Supplementary material 4

### S4. Results for the sensitivity analysis comparing XGBoost Logistic Regression and Deep Neuronal Neworks

Sensitivity analysis: XGBoost model performance compared to Logistic Regression and Deep Neural Networks using gradients

The three ML models - logistic regression, XGBoost and DNN - achieved comparable AUROC predictive performance in a 5-fold cross-validation setup for suboptimal responders resulting in COVID-19 related mortality, hospitalisation, and ICU admissions. XGBoost reported a slightly higher performance overall (Table 3). Similar predictors were identified across the different models (S1 Appendix 5. Sensitivity analysis for the Logistic regression model and S1 Appendix 6. Sensitivity analysis with Deep Neural Networks using gradients)

Table 3 - Performance of different models for predicting suboptimal response cases resulting COVID-19 related mortality, hospitalisation and ICU admissions.

| **Outcome categories** | **Models** | **AUROC** |
| --- | --- | --- |
| **Mortality prediction** | Logistic Regression | 0.8748 ± 0.0102 |
|  | XG Boost | **0.9256 ± 0.0125** |
|  | DNN | 0.9086 ± 0.0312 |
| **Hospitalization prediction** | Logistic Regression | 0.8088 ± 0.0057 |
|  | XG Boost | **0.8198 ± 0.0185** |
|  | DNN | 0.8095 ± 0.0115 |
| **ICU admission** | Logistic Regression | 0.7975 ± 0.0215 |
|  | XG Boost | **0.8329 ± 0.0119** |
|  | DNN | 0.824 ± 0.035 |

AUROC: Area under the receiver operating characteristic curve.
