# Supplementary material for: Prediction of COVID-19 hospitalisation, ICU admission or death following ChAdOx1 vaccination using artificial intelligence: A clinical predictive model from the English RAVEN study
Source: PLoS One. 2026 Feb 20;21(2):e0336449. doi: 10.1371/journal.pone.0336449 (PMC12923009; doi:10.1371/journal.pone.0336449)
Supplement: S1 File — S1. Comorbidities based on the (COVID-19) green book Chapter 14a definitions. S2. Cambridge Multimorbidity Score. S3. Algorithm defining COVID-19 vaccination. S4. Results for the sensitivity analysis comparing XGBoost Logistic Regression and Deep Neuronal Neworks. S5. Sensitivity analysis for the Logistic regression model. S6. Sensitivity analysis with Deep Neural Networks using gradients. S7. Tables with the coefficients of the logistic regression trained for predicting the breakthrough cases leading to mortality. S8. Tables with the coefficients of the logistic regression trained for predicting the breakthrough cases leading to hospitalisation. S9. Tables with the coefficients of the logistic regression trained for predicting the breakthrough cases leading to ICU admission. S10. Tables with the SHAP values highlighting the relevance of different input variables in XGBoost trained for predicting breakthrough cases resulting in mortality. S11. Tables with the SHAP values highlighting the relevance of different input variables in XGBoost trained for predicting breakthrough cases resulting in hospitalisation. S12. Tables with the SHAP values obtained from XGBoost trained for the ICU admission prediction. (ZIP) [file pone.0336449.s001.zip › S3_RAVEN_AI_20260205.docx]

Supplementary material 3

### S3. Algorithm defining COVID-19 vaccination

The steps are as follows:

1. Find vaccinations data and prioritise NIMS data over ORCHID data
2. Define a “valid” vaccination as
   1. A vaccination which has a brand recorded.
   2. For two doses, there needs to be a minimum interval between the doses
      1. The shortest gap listed for Pfizer vaccination is 21 days apart. <https://www.gov.uk/government/publications/regulatory-approval-of-pfizer-biontech-vaccine-for-covid-19/information-for-uk-recipients-on-pfizerbiontech-covid-19-vaccine>
      2. The shortest gap for the ChAdOx1 COVID-19 vaccine is 4 weeks apart. <https://www.gov.uk/government/news/statement-from-the-uk-chief-medical-officers-on-the-prioritisation-of-first-doses-of-covid-19-vaccines#:~:text=The%20MHRA%20authorisation%20includes%20conditions,weeks%20after%20the%20first./>
   3. For three doses, there needs to be a minimum interval between the doses
      1. The shortest gap listed for Pfizer vaccination is 8 weeks after the second injection. <https://www.gov.uk/government/publications/regulatory-approval-of-pfizer-biontech-vaccine-for-covid-19/information-for-uk-recipients-on-pfizerbiontech-covid-19-vaccine>
      2. The shortest gap for the ChAdOx1 COVID-19 vaccine is 4 weeks after the second dose
3. Exclude duplicate entries. Where there are two vaccine administration dates within 14 days, take the earlier date.
4. Classify into combinations of two vaccine doses: Pfizer-Pfizer, ChAdOx1 – ChAdOx1 and other vaccine combinations.
