# Supplementary material for: Prediction of COVID-19 hospitalisation, ICU admission or death following ChAdOx1 vaccination using artificial intelligence: A clinical predictive model from the English RAVEN study
Source: PLoS One. 2026 Feb 20;21(2):e0336449. doi: 10.1371/journal.pone.0336449 (PMC12923009; doi:10.1371/journal.pone.0336449)
Supplement: S1 File — S1. Comorbidities based on the (COVID-19) green book Chapter 14a definitions. S2. Cambridge Multimorbidity Score. S3. Algorithm defining COVID-19 vaccination. S4. Results for the sensitivity analysis comparing XGBoost Logistic Regression and Deep Neuronal Neworks. S5. Sensitivity analysis for the Logistic regression model. S6. Sensitivity analysis with Deep Neural Networks using gradients. S7. Tables with the coefficients of the logistic regression trained for predicting the breakthrough cases leading to mortality. S8. Tables with the coefficients of the logistic regression trained for predicting the breakthrough cases leading to hospitalisation. S9. Tables with the coefficients of the logistic regression trained for predicting the breakthrough cases leading to ICU admission. S10. Tables with the SHAP values highlighting the relevance of different input variables in XGBoost trained for predicting breakthrough cases resulting in mortality. S11. Tables with the SHAP values highlighting the relevance of different input variables in XGBoost trained for predicting breakthrough cases resulting in hospitalisation. S12. Tables with the SHAP values obtained from XGBoost trained for the ICU admission prediction. (ZIP) [file pone.0336449.s001.zip › S2_RAVEN_AI_20260205.docx]

Supplementary material 2

### S2. Cambridge Multimorbidity Score

The original Cambridge Multimorbidity Score (CMMS) (22) was based on 37 conditions and 20 in the reduced model. An updated CMMS score using ORCHID data was based on 21 of the 37 conditions (57). For the analysis of GDPPR data, a validated version of CMMS will be used, from a full model of 22 conditions (for which the match between the ORCHID data and GDPPR data was high, measured by sensitivity>80%, or sensitivity is low but the condition has high prognostic value) of which 17 are retained in the final model.

**CMMS conditions**

| Condition | Payne 2020  37 items | Payne 2020  20 items | Updated  ORCHID  final model  21 items | Updated  GDPPR  full model  22 items | Updated GDPPR  final model  17 items |
| --- | --- | --- | --- | --- | --- |
| Alcohol problems | x | x | x | x | x |
| Anorexia or bulimia | x |  |  |  |  |
| Anxiety or depression | x | x | x |  |  |
| Asthma | x | x |  | x |  |
| Atrial fibrillation | x | x | x | x | x |
| Blindness & low vision | x |  |  |  |  |
| Bronchiectasis | x |  |  | x |  |
| Cancer | x | x | x | x | x |
| Chronic kidney disease | x | x | x | x | x |
| Chronic liver disease & viral hepatitis | x |  | x | x | x |
| Chronic sinusitis | x |  |  | x |  |
| Connective tissue disorder* | x | x |  |  |  |
| Constipation | x | x | x | x | x |
| COPD | x | x | x | x | x |
| Coronary heart disease | x | x |  | x | x |
| Dementia | x | x | x | x | x |
| Diabetes | x | x | x | x | x |
| Disorder of prostate |  |  | x |  |  |
| Diverticular disease of intestine | x |  |  |  |  |
| Epilepsy | x | x | x | x | x |
| Hearing loss | x | x |  |  |  |
| Heart failure | x | x | x | x | x |
| Hypertension | x | x |  | x |  |
| Inflammatory bowel disease | x |  |  |  |  |
| Irritable bowel syndrome | x | x | x |  |  |
| Learning disability | x |  | x | x | x |
| Migraine | x |  |  |  |  |
| Multiple sclerosis | x |  | x |  |  |
| Painful condition | x | x | x |  |  |
| Parkinsonism | x |  | x | x | x |
| Peptic ulcer disease | x |  |  |  |  |
| Peripheral vascular disease | x |  | x | x | x |
| Psoriasis or eczema | x |  |  |  |  |
| Psychoactive substance misuse | x |  | x |  |  |
| Psychosis or bipolar disorder** | x | x | x | x | x |
| Stroke & TIA | x | x |  | x | x |
| Thyroid disorders | x |  |  | x |  |

COPD – Chronic obstructive pulmonary disease, TIA – Transient ischaemic attack

For RAVEN study: *Lists as Rheumatoid arthritis;** Lists as Schizophrenia or bipolar disorder;

**Conditions and weights**

| **Condition** | **Basis of definition*** | **Weight** |
| --- | --- | --- |
| Alcohol problems | SNOMED CT ever recorded | 0.5670 |
| 0.2779Atrial fibrillation | SNOMED CT ever recorded | 0.2779 |
| 0.1286Cancer (excl non melanoma) | SNOMED CT (first) recorded in last 5 years | 1.1876 |
| Chronic kidney disease | Highest value of last 2 eGFR readings is <60 ml/min | 0.1286 |
| Chronic liver disease and viral hepatitis | SNOMED CT ever recorded | 1.0844 |
| 0.1201Constipation | ≥4 laxative prescriptions | 0.5889 |
| COPD | SNOMED CT ever recorded | 0.6638 |
| Coronary heart disease | SNOMED CT ever recorded | 0.1201 |
| Dementia | SNOMED CT ever recorded | 0.9815 |
| Diabetes | SNOMED CT ever recorded | 0.2623 |
| Epilepsy | SNOMED CT AND ≥1 antiepileptic prescription | 0.6714 |
| Heart failure | SNOMED CT ever recorded | 0.5022 |
| 0.5621Learning disability | SNOMED CT ever recorded | 1.0992 |
| Parkinsonism | SNOMED CT ever recorded | 0.5339 |
| Peripheral vascular disease | SNOMED CT ever recorded | 0.3519 |
| Schizophrenia or bipolar disorder | SNOMED CT ever recorded OR lithium ever prescribed | 0.5621 |
| Stroke and transient ischaemic attack | SNOMED CT ever recorded | 0.2299 |

COPD – Chronic obstructive pulmonary disease, eGFR - estimated glomerular filtration rate

* For all medicines, in the last 12 months, unless otherwise stated
