# Supplementary material for: Prediction of COVID-19 hospitalisation, ICU admission or death following ChAdOx1 vaccination using artificial intelligence: A clinical predictive model from the English RAVEN study
Source: PLoS One. 2026 Feb 20;21(2):e0336449. doi: 10.1371/journal.pone.0336449 (PMC12923009; doi:10.1371/journal.pone.0336449)
Supplement: S1 File — S1. Comorbidities based on the (COVID-19) green book Chapter 14a definitions. S2. Cambridge Multimorbidity Score. S3. Algorithm defining COVID-19 vaccination. S4. Results for the sensitivity analysis comparing XGBoost Logistic Regression and Deep Neuronal Neworks. S5. Sensitivity analysis for the Logistic regression model. S6. Sensitivity analysis with Deep Neural Networks using gradients. S7. Tables with the coefficients of the logistic regression trained for predicting the breakthrough cases leading to mortality. S8. Tables with the coefficients of the logistic regression trained for predicting the breakthrough cases leading to hospitalisation. S9. Tables with the coefficients of the logistic regression trained for predicting the breakthrough cases leading to ICU admission. S10. Tables with the SHAP values highlighting the relevance of different input variables in XGBoost trained for predicting breakthrough cases resulting in mortality. S11. Tables with the SHAP values highlighting the relevance of different input variables in XGBoost trained for predicting breakthrough cases resulting in hospitalisation. S12. Tables with the SHAP values obtained from XGBoost trained for the ICU admission prediction. (ZIP) [file pone.0336449.s001.zip › S1_RAVEN_AI_20260205.docx]

Supplementary material S1

### S1. Comorbidities based on the (COVID-19) green book Chapter 14a definitions

| **Risk Group** | **Green book definition** |
| --- | --- |
| Chronic respiratory disease | Individuals with a severe lung condition, including those with poorly controlled asthma that requires continuous or repeated use of systemic steroids or with previous exacerbations requiring hospital admission, and chronic obstructive pulmonary disease (COPD) including chronic bronchitis and emphysema; bronchiectasis, cystic fibrosis, interstitial lung fibrosis, pneumoconiosis and bronchopulmonary dysplasia (BPD). |
| Chronic heart disease and vascular disease | Congenital heart disease, hypertension with cardiac complications, chronic heart failure, individuals requiring regular medication and/or follow-up for ischaemic heart disease. This includes individuals with atrial fibrillation, peripheral vascular disease or a history of venous thromboembolism. |
| Chronic kidney disease | Chronic kidney disease at stage 3, 4 or 5, chronic kidney failure, nephrotic syndrome, kidney transplantation |
| Chronic liver disease | Cirrhosis, biliary atresia, chronic hepatitis |
| Chronic neurological disease | Stroke, transient ischaemic attack (TIA). Conditions in which respiratory function may be compromised due to neurological or neuromuscular disease (e.g. polio syndrome sufferers). This group also includes individuals with cerebral palsy, severe or profound and multiple learning disabilities (PMLD), Down’s syndrome, multiple sclerosis, epilepsy, dementia, Parkinson’s disease, motor neurone disease and related or similar conditions; or hereditary and degenerative disease of the nervous system or muscles; or severe neurological disability. |
| Diabetes mellitus and other endocrine disorders | Any diabetes, including diet-controlled diabetes, current gestational diabetes, and Addison's disease. |
| Immunosuppression | Immunosuppression due to disease or treatment, including patients undergoing chemotherapy leading to immunosuppression, patients undergoing radical radiotherapy, solid organ transplant recipients, bone marrow or stem cell transplant recipients, Human immunodeficiency virus (HIV) infection at all stages, multiple myeloma or genetic disorders affecting the immune system (e.g. IRAK-4, NEMO, complement disorder, Severe combined immunodeficiency - SCID).Individuals who are receiving immunosuppressive or immunomodulating biological therapy including, but not limited to, anti-TNF, alemtuzumab, ofatumumab, rituximab, patients receiving protein kinase inhibitors or PARP inhibitors, and individuals treated with steroid sparing agents such as cyclophosphamide and mycophenolate mofetil. Individuals treated with or likely to be treated with systemic steroids for more than a month at a dose equivalent to prednisolone at 20mg or more per day for adults. Anyone with a history of haematological malignancy, including leukaemia, lymphoma, and myeloma and those with systemic lupus erythematosus and rheumatoid arthritis, and psoriasis who may require long term immunosuppressive treatments |
| Asplenia or dysfunction of the spleen | This also includes conditions that may lead to splenic dysfunction, such as homozygous sickle cell disease, thalassemia major and coeliac syndrome |
| Morbid obesity | Adults with a Body Mass Index (BMI) ≥40 kg/m² |
| Severe mental illness | Individuals with schizophrenia or bipolar disorder, or any mental illness that causes severe functional impairment. |
| Younger adults in long-stay nursing and residential care settings | Many younger adults in residential care settings will be eligible for vaccination because they fall into one of the clinical risk groups above (for example learning disabilities). |

Provided by University of Nottingham PRIMIS
